# A Method for Confidence Intervals of High Quantiles

**DOI:** 10.3390/e23010070

**Published:** 2021-01-04

**Authors:** Mei Ling Huang, Xiang Raney-Yan

**Affiliations:** 1 Department of Mathematics, Brock University, St. Catharines, ON L2S 3A1, Canada; 2 Department of Mathematcs, Niagara College, Welland, ON L3C 7L3, Canada; xyan@niagaracollege.ca

**Keywords:** efficiency, extreme value distributions, generalized Pareto distribution, Hill estimator, mean square errors, order statistics, tail index, Weissman estimator

## Abstract

The high quantile estimation of heavy tailed distributions has many important applications. There are theoretical difficulties in studying heavy tailed distributions since they often have infinite moments. There are also bias issues with the existing methods of confidence intervals (CIs) of high quantiles. This paper proposes a new estimator for high quantiles based on the geometric mean. The new estimator has good asymptotic properties as well as it provides a computational algorithm for estimating confidence intervals of high quantiles. The new estimator avoids difficulties, improves efficiency and reduces bias. Comparisons of efficiencies and biases of the new estimator relative to existing estimators are studied. The theoretical are confirmed through Monte Carlo simulations. Finally, the applications on two real-world examples are provided.

## 1. Introduction

Extreme value analysis (EVA) was first introduced by Leonard Tippett (Fisher and Tippett, 1928 [1]). Tippett was working on how to make cotton thread stronger, he realized that the strength of the weakest threads were the only factor that matters when it comes to deciding the strength of the cotton thread. Nowadays, extreme value analysis is widely used in almost all fields, from engineering, social science, economics, traffic predictions to insurance and so on. People are interested in extreme events in these fields such as, the shortest life span of a new engine, the maximum appreciation of the stock market, the longest driving time on a highway at rush hour, or the biggest medical claim to an insurance company. The distributions of these extreme events are usually unknown. In general, EVA involves the extrapolation of an unknown distribution and its high quantiles. Estimating high quantile based on observation is very important in EVA, since it gives the corresponding value *x* for a very small exceeding possibility *p*.

There are certain risks, ones that are not decided by us or can barely be predicted until right before they are about to happen. This can include things such as an earthquake, terrorist attacks, a virus breakout, and so forth. For these events, we will need risk management which is in place to minimize, monitor, and control the impact of unfortunate events, or to maximize the realization of opportunities. Estimating the confidence interval of high quantiles plays an important role in risk management. Since a high quantile is located at the tail area, it heavily depends on the behaviour of the tail distribution, or from the statistical point of view, it depends on the *k* largest order statistics. This leads to the challenges of the instability in the choice of k, and the bias issues. There are many research on the mathematical models and theoretical studies in the literature for estimating confidence intervals of high quantiles, we review them in Section 2.

This paper proposes a new method to estimate high quantile of a heavy-tailed distribution. The new method has interesting improvements compared with other existing methods. This paper makes three main contributions to methodology.

(1) This paper proposes a new estimation method based on a geometric mean with good asymptotic properties. It is consistent and stable relative to the existing methods. The paper provides a computational algorithm which overcomes the mathematical difficulties and bias problems of the estimation of confidence intervals of high quantiles of a heavy tailed distribution.

(2) The Monte Carlo simulation studies on three heavy tailed distribution models: Fréchet (0.25), GPD (0.5) and GPD(2) (GPD: generalized Pareto distribution). The simulation results confirm that the proposed method is more efficient relative to the existing quantile estimators.

(3) This paper uses the proposed estimation method to predict extreme values in the flu in Canada, and gamma ray from solar flare examples. It is interesting to see that these data sets fit the GPD model very well. We apply the proposed method to estimate the confidence intervals of high quantiles. The numerical results show that the proposed method gives more efficient results compared with other existing methods.

In this paper, we review several existing high quantile estimators with their behavior in Section 2. We propose a new estimator for the confidence interval of high quantiles based on the geometric mean and explore its asymptotic properties in Section 3. To compare the new estimator with the existing estimators, Section 4 presents Monte Carlo simulation results and the improvement of the proposed quantile estimator relative to existing methods. In Section 5 we apply the proposed new method to construct confidence intervals of high quantiles on flu in Canada and gamma ray examples. Finally, conclusions and discussions are given in Section 6.

## 2. Existing Estimator for High Quantiles

Heavy-tailed distributions (de Haan and Ferreira, 2006 [2]) is important to extreme value events.

**Definition** **1.** *A random variable X is said to have a heavy tail distribution if its distribution function F(x) satisfies*

$$1-F\left(x\right)=L\left(x\right)x^{-1/\gamma },\;x\in \left(-\infty ,\infty \right),\;\mathrm{as}\;x\to \infty ,\;\gamma >0,$$

*where*L(t)*is a slowly varying function*$with\lim_{t\to \infty }\frac{L(tx)}{L(t)}=1,$ for all x>0. γ *is the tail index.*

Notice that we can have $L\left(x\right)={\left(\ln\left(x\right)\right)}^{b},$ b∈ℝ (de Hann and Ferreira, 2006, p. 362 [2]). Since L(t) behaves approximately as a constant *c*, for simplicity, we assume that a heavy tailed distribution satisfies

(1) $$1-F\left(x,\gamma \right)\to cx^{-1/\gamma },\;x\in \left(-\infty ,\infty \right),\;\mathrm{as}\;x\to \infty ,\;c>0,\;\gamma >0.$$

Since the heavy tailed distributions decay slower than the exponential distributions and have longer tails. A tail function is defined as

**Definition** **2.** *A tail function U(t) of any distribution function F(x) is defined as*

$$U\left(t\right)={\left(\frac{1}{1-F}\right)}^{⟵},\;\;\;where\;“{\;}^{⟵}\;”\;denotes\;the\;inverse\;function.$$

For the heavy tailed distribution in (1), we can rewrite the tail function as

(2) $$U\left(t\right)={\left(\frac{1}{ct^{-1/\gamma }}\right)}^{⟵}=c^{\gamma }t^{\gamma }=Ct^{\gamma },\;\mathrm{as}\;t\to \infty ,\;\;\mathrm{where}\;c^{\gamma }={\left(L\left(t\right)\right)}^{\gamma },\;\mathrm{let}\;C=c^{\gamma }.$$

**Definition** **3.** *The quantile function Q(1−p,γ) of a heavy tailed distribution F(x,γ) in (1) for a given probability 1−p is defined by*

$$x_{1-p}=Q\left(1-p,\gamma \right)=\inf\left\{x:F(x,\gamma )\geq 1-p\right\},\;x\in \left(-\infty ,\infty \right),\;0<p<1$$

*where Q(1−p,γ) is the generalized inverse function of F, we call Q(1−p,γ) the (1−p)th quantile function of F(x).*

Value at Risk (VaR) is widely used in risk management. When *p* is very small, $x_{1-p}$ becomes a high quantile as the *p*th value at risk, we define

(3) $$VaR_{p,\gamma }=x_{1-p}=Q\left(1-p,\gamma \right),\;0<p<1,\;p\;\mathrm{is}\;\mathrm{very}\;\mathrm{small}.$$

Also we can use the tail function in (2) to write $VaR_{p,\gamma }$ as

$$VaR_{p,\gamma }=U\left(\frac{1}{p},\gamma \right)=Ct^{\gamma },\;t=\frac{1}{p},\;p=p_{n}\to 0,\;np_{n}\to 0,\;as\;n\to \infty .$$

The heavy-tailed models have a compulsory infinite right endpoint. In the case of negative observations in the model, the sample size should be exclusively the number of positive observations, $n^{+},$ although a deterministic shift in the data is preferred by some authors, to work only with positive values. In this paper, we use the real line (0,∞).

To estimate $VaR_{p,\gamma },$ let $X_{1:n}\leq X_{2:n}\leq \;...\;\leq X_{n:n}$ be the order statistics from a random sample $X_{1},X_{2},...X_{n}.$ We review the four high quantile estimation methods in the literature.

### 2.1. Quantile Function-Tail Index Method

For estimating high quantiles, we use the ln function, and estimate the tail index first

(4) $$\ln Q_{\hat{\gamma }}^{\left(p\right)}=\ln VaR_{p,\hat{\gamma }}=\ln x_{1-p,\hat{\gamma }},\;\;0<p<1,\;p\;\mathrm{is}\;\mathrm{very}\;\mathrm{small}.$$

To estimate high quantile function, we estimate the tail index first (Dekkers and de Haan 1989 [3]). Hill (1975) [4] estimator is a well known consistent estimator for tail index γ.

**Definition** **4.** *Consider the order statistics $X_{n-k:n}$, and k as an intermediate sequence of integers, Hill estimator is defined as*

(5) $${\hat{\gamma }}_{H}=H\left(k\right)=\frac{1}{k}\sum_{i=1}^{k}U_{i},\;U_{i}=i\left(\ln\frac{X_{n-i+1:n}}{X_{n-i:n}}\right),\;1\leq i\leq k.$$

*where*$k=k_{n}\to \infty$, k∈[1,n), k=o(n)asn→∞.

The Hill estimator ${\hat{\gamma }}_{H}=H\left(k\right)$ in (5) used largest *k* order statistics of a random sample. Substitute ${\hat{\gamma }}_{H}=H\left(k\right)$ defined in (5) into (4), then we obtain ln (1−p)th high quantile as

(6) $$\ln q_{H,p}\left(k\right)=\ln Q_{H}^{\left(p\right)}\left(k\right)=\ln x_{1-p,H}\left(k\right),\;0<p<1,\;p\;\mathrm{is}\;\mathrm{very}\;\mathrm{small}.$$

This estimator depends on k, small values of *k* provide high volatility whereas large values of *k* induce considerable bias. Hence, semi-parametric extensions may be considered for increasing the degree of freedom in the trade-off between variance and bias. Note that the tail index γ is a parameter of a given distribution, and a quantile of a distribution is a function of γ.

### 2.2. Weissman Method

Weissman (1978) [5] proposed the following semiparametric estimator of a high quantile

$$Q_{\hat{\gamma }}^{\left(p\right)}\left(k\right)=VaR_{p,\hat{\gamma }}=x_{1-p,\hat{\gamma }}=X_{n-k:n}{\left(\frac{k}{np}\right)}^{\hat{\gamma }},\;0<p<1,\;1\leq k\leq n-1,\;\;\mathrm{and}$$

$$\ln Q_{\hat{\gamma }}^{\left(p\right)}\left(k\right)=\ln X_{n-k:n}+\hat{\gamma }\ln\left(\frac{k}{np}\right).$$

We substitute ${\hat{\gamma }}_{H}=H\left(k\right)$ in (5) into the function above, then we have,

(7) $$\ln{\hat{Q}}_{H}^{\left(p\right)}\left(k\right)=\ln X_{n-k:n}+H\left(k\right)\ln\left(\frac{k}{np}\right),\;1\leq k\leq n-1.$$

Without any prior indication on *k*, the Weissman estimator shows a large volatility as it depends on the fraction sample *k*. Although the minimization of the bias and MSE can be considered as a criterion to select *k*, it is impractical as they are unknown. Other methods for the selection of sample fraction *k* can be found in Beirlant et al. (1996) [6]; Dreea and Kaufmann (1998) [7]; Guillou and Hall (2001) [8]; Gomes and Oliveira (2001) [9].

The optimal *k* value through the tail index Hill estimator H, $k_{0},$ is given by formula (15) in Section 2.4 Optimal *k* Values.

### 2.3. Reduced-Bias Method

Hall and Welsh (1985) [10] proposed a second-order expansion on the tail function *U* in (2)

(8) $$U\left(t\right)=Ct^{\gamma }\left(1+\frac{A(t)}{\rho }+o\left(t^{\rho }\right)\right),\;A\left(t\right)=\gamma \beta t^{\rho },\;as\;t\to \infty ,$$

with C, γ>0, ρ<0, and β ≠0. Where β is the scale second-order parameter and ρ is the shape second-order parameter.

To further reduce the bias of quantile estimators which requires us to observe the behavior of the estimation of the second-order parameters β and ρ. Second-order reduced-bias was discussed by Peng (1998) [11], Beirlant, Dierckx, Goegebeur and Mattys (1999) [12], Freueverger and Hall (1999) [13], Gomes, Martins and Neves (2000) [14], Caeiro and Gomes (2002) [15], Gomes, Figueiredo and Mendonea (2004) [16], among others. Comes and Pestana (2007) [17] considered the estimators $\left(\hat{\rho_{\tau }}\left(k\right),\hat{\beta_{\hat{\rho }}}\left(k\right)\right)$ for the second-order parameters (ρ,β).

Careiro et al. (2005, p. 122) [18] advises the the use of turning parameter τ in the estimation of ρ. It provides higher stability as functions of k, the number of the top order statistics used, for a wide range of large *k* value, by means of any stability criterion.

**Definition** **5.** *Caeiro et al. (2005) [18] defined the bias-corrected Hill estimator*

(9) $$\bar{H}\left(k\right)\equiv {\bar{H}}_{\hat{\beta },\hat{\rho }}\left(k\right)=H\left(k\right)\left(1-\frac{\hat{\beta }}{1-\hat{\rho }}{\left(\frac{n}{k}\right)}^{\hat{\rho }}\right),$$

*where*H(k)*is defined in (5). For a tuning real parameter τ∈ℝ,*

(10) $$\hat{\rho_{\tau }}\left(k\right)\equiv {\hat{\rho_{n}}}^{\left(\tau \right)}\left(k\right)=\min\left(0,\frac{3\left(T_{n}^{\left(\tau \right)}\left(k\right)-1\right)}{T_{n}^{\left(\tau \right)}\left(k\right)-3}\right)$$

$$T_{n}^{\left(\tau \right)}\left(k\right)=\left\{\begin{array}{c} \frac{{\left(M_{n}^{\left(1\right)}\left(k\right)\right)}^{\tau }-{\left(\frac{M_{n}^{\left(2\right)}\left(k\right)}{2}\right)}^{\frac{\tau }{2}}}{{\left(\frac{M_{n}^{\left(2\right)}\left(k\right)}{2}\right)}^{\frac{\tau }{2}}-{\left(\frac{M_{n}^{\left(3\right)}\left(k\right)}{6}\right)}^{\frac{\tau }{3}}}\;\;\;\;\mathrm{if}\;\tau \neq 0;\\ \frac{\ln\left(M_{n}^{\left(1\right)}\left(k\right)\right)-\frac{1}{2}\ln\left(\frac{M_{n}^{\left(2\right)}\left(k\right)}{2}\right)}{\frac{1}{2}\ln\left(\frac{M_{n}^{\left(2\right)}\left(k\right)}{2}\right)-\frac{1}{3}\ln\left(\frac{M_{n}^{\left(3\right)}\left(k\right)}{6}\right)}\;\mathrm{if}\;\tau =0, \end{array}\right.$$

$$M_{n}^{\left(j\right)}\left(k\right)=\frac{1}{k}\sum_{i=1}^{k}{\left(\log X_{n-i+1:n}-\log X_{n-k:n}\right)}^{j},\;j=1,2,3.$$

(11) $$\hat{\beta_{\hat{\rho }}}\left(k\right)={\left(\frac{k}{n}\right)}^{\hat{\rho }}\frac{d_{\hat{\rho }}\left(k\right)D_{0}\left(k\right)-D_{\hat{\rho }}\left(k\right)}{d_{\hat{\rho }}\left(k\right)D_{\hat{\rho }}\left(k\right)-D_{\hat{2\rho }}\left(k\right)},$$

$$where\;for\;any\;\theta \leq 0,\;\;d_{\theta }\left(k\right)=\frac{1}{k}\sum_{i=1}^{k}{\left(\frac{i}{k}\right)}^{-\theta }\;\mathrm{and}\;D_{\theta }\left(k\right)=\frac{1}{k}\sum_{i=1}^{k}{\left(\frac{i}{k}\right)}^{-\theta }U_{i},$$

*with*$U_{i}$*as defined in (5) that*1≤i≤k, *and (10) achieves consistency if*$\sqrt{k}A\left(n/k\right)\to \infty$*as*n→∞*and*$\hat{\rho }-\rho =o_{\rho }\left(1/\ln n\right)$.

The corresponding ln-quantile estimator with the tail index estimator $\bar{H}$ in (9) is

(12) $$\ln{\hat{Q}}_{\bar{H}}^{\left(p\right)}\left(k\right)=\ln X_{n-k+1:n}+\bar{H}\left(k\right)\ln\left(\frac{k}{np}\right),\;1\leq k\leq n-1.$$

A similar estimator to the estimator in (12) is considered in Lekina et al. (2014) [19] and Lekina (2010) [20].

Gomes and Pestana (2007) [17] considered the ln-Var estimator

(13) $$\ln{\bar{Q}}_{\hat{\gamma }}^{\left(p\right)}\left(k\right)=\ln X_{n-k+1:n}+\hat{\gamma }\left(\ln\left(\frac{k}{np}\right)+C_{p}\left(k;\hat{\beta },\hat{\rho }\right)\right),\;C_{p}\left(k;\hat{\beta },\hat{\rho }\right)=\hat{\beta }{\left(\frac{n}{k}\right)}^{\hat{\rho }}\frac{{\left(\frac{k}{np}\right)}^{\hat{\rho }}-1}{\hat{\rho }}.$$

Substitute the estimator $\bar{H}$ in (9) into (13), we have another estimator for high quantile as

(14) $$\ln{\bar{Q}}_{\bar{H}}^{\left(p\right)}\left(k\right)=\ln X_{n-k+1:n}+\bar{H}\left(k\right)\left(\ln\left(\frac{k}{np}\right)+C_{p}\left(k;\hat{\beta },\hat{\rho }\right)\right).$$

### 2.4. Optimal k Values

As discussed previously, we have problem that the estimation varies as the *k* varies, and it become very unreliable when *k* is large. Gomes and Pestana (2007) [17] suggested to use the numerically estimated optimal *k* values.

The optimal *k* for the tail index estimator through Hill estimator H(k) in (5) is $k_{0},$

(15) $$k_{0}={\left(\frac{\left(1-\rho \right)n^{-\rho }}{\beta \sqrt{-2\rho }}\right)}^{\frac{2}{1-2\rho }}.$$

The optimal *k* for the semiparametric quantile estimator $\ln Q_{H}\left(k\right)$ in (7), is $k_{0}^{Q_{H}},$

(16) $$k_{0}^{Q_{H}}=\underset{k}{\arg\min}\left\{\ln^{2}\left(\frac{k}{np}\right)\left(\frac{1}{k}+\frac{\beta^{2}{\left(n/k\right)}^{2\rho }}{{\left(1-\rho \right)}^{2}}\right)\right\}.$$

The optimal *k* for the second-order reduced-bias quantile estimator $\ln Q_{\bar{H}}^{\left(p\right)}\left(k\right)$ in (12) and $\ln{\bar{Q}}_{\bar{H}}^{\left(p\right)}\left(k\right)$ in (14) should be larger than $k_{0}$, is $k_{01}$,

(17) $$k_{01}={\left(\frac{1.96\left(1-\rho \right)n^{-\rho }}{\left|\beta \right|}\right)}^{\frac{2}{1-2\rho }}.$$

By using these optimal *k* values, all the quantile estimators provide better results. However, with an unknown distribution, and estimated second-order parameters, these numerically estimated *k* values are not always accurate. Since all the quantile estimators are so sensitive to the *k* value, in this paper, we propose a new quantile estimator which does not depends on k.

## 3. New Estimator for High Quantile

### 3.1. New Estimator

Our goal is to improve the quantile estimators in Section 2. There are bias issues and difficult in determining *k* with the existing estimating methods. In order to overcome these problems, Huang (2011) [21] proposed a new quantile estimator which is the geometric mean of the reduced-bias quantile estimator in (14).

**Definition** **6.** (18) $${\hat{Q}}_{New,\hat{\gamma }}^{\left(p\right)}={\left[\prod_{k=1}^{n-1}\left(X_{n-k+1:n}{\left(\frac{k}{np}\right)}^{\hat{\gamma }}\right)\right]}^{\frac{1}{n-1}},\;0<p<1,\;1\leq k\leq n-1.$$

*where* $X_{n-k:n}$ *is the* (k+1)th *top order statistic,* $\hat{\gamma }$ *is any consistent estimator for γ, and Q stands for quantile function.
*

Based on (16), (20) can be written as

(19) $$\ln{\hat{Q}}_{New,\hat{\gamma }}^{\left(p\right)}=\frac{1}{n-1}\sum_{k=1}^{n-1}\left[\ln X_{n-k+1:n}+\hat{\gamma }\left(\ln\left(\frac{k}{np}\right)+\alpha C_{p}\left(k;\hat{\beta },\hat{\rho }\right)\right)\right],$$

where 0<p<1 and α is a constant that α∈ℝ.

$\alpha C_{p}\left(k;\hat{\beta },\hat{\rho }\right)$ is the adjustment term, where $C_{p}\left(k;\hat{\beta },\hat{\rho }\right)$ is defined in (13) that reduces bias using the second-order parameters. α is a key value depends only on *n* to furthermore reduce the bias by observing the behavior of the second-order parameters. We will discuss the choice of α in Section 4.

Section 3, Section 4 and Section 5 will show that the new estimator $\ln{\hat{Q}}_{New,\hat{\gamma }}^{\left(p\right)}$ has good properties, and

1. The new quantile estimator $\ln{\hat{Q}}_{New,\hat{\gamma }}^{\left(p\right)}$ has the least bias, the smallest MSE and the highest efficiency.

2. The new quantile estimator $\ln{\hat{Q}}_{New,\hat{\gamma }}^{\left(p\right)}$ is consistent and does not depend on *k* as the existing quantile estimators does.

3. The confidence interval based on the new quantile estimator $\ln{\hat{Q}}_{New,\hat{\gamma }}^{\left(p\right)}$ is the most efficient compared to the existing methods, where it not only has the shortest length of the interval, but also has the highest probability coverage of the true value in most cases.

### 3.2. Asymptotic Properties of the New Estimator ln${\hat{Q}}_{New,\bar{H}}^{\left(p\right)}$

Using the Hall-Welsh class of model in (8), we derive that the new estimator $\ln{\hat{Q}}_{New,\hat{\gamma }}^{\left(p\right)}$ in (19) has the asymptotic properties under following conditions, when $\hat{\gamma }=\bar{H}$ in (5).

**Condition 1** **(C1).** *For intermediate k,$k=k_{n}\to \infty .$k∈[1,n),k=o(n), as n→∞.*

**Condition 2** **(C2).** *$\ln\left(np_{n}\right)=o\left(\sqrt{k}\right),\;\lim_{n\to \infty }\sqrt{k}A\left(\frac{n}{k}\right)=\lambda \in \mathbb{R},$ where A is in (8).*

**Theorem** **1.** *Under (C1) and (C2), if we use $\hat{\gamma }=\bar{H}$* in (5), then $ln{\hat{Q}}_{New,\bar{H}}^{\left(p\right)}$ has a asymptotic normal distribution

(20) $$\left(\ln{\hat{Q}}_{New,\bar{H}}^{\left(p\right)}-\ln VaR_{p}\right)\overset{d}{\underset{n\to \infty }{⟶}}Normal\left(0,\frac{\gamma^{2}}{{\left(n-1\right)}^{2}}\left[\sum_{k=1}^{n-1}\frac{{\left[\ln\frac{k}{np}\right]}^{2}}{k}+w\underset{i<j}{\sum^{n-1}\sum^{n-1}}\left[\frac{\ln\left(\frac{i}{np}\right)\ln\left(\frac{j}{np}\right)}{\sqrt{i\cdot j}}\right]\right]\right).$$

*The asymptotic mean, variance and efficiency of $\ln{\hat{Q}}_{New,\bar{H}}^{\left(p\right)}\left(k\right)$ in (19) relative to $\ln{\bar{Q}}_{\bar{H}}^{\left(p\right)}\left(k\right)$ in (14) are given by*

$$E\left(\ln{\hat{Q}}_{New,\bar{H}}^{\left(p\right)}\right)\underset{n\to \infty }{\approx }\ln VaR_{p};$$

(21) $$\begin{array}{c} Var\left(\ln{\hat{Q}}_{New,\bar{H}}^{\left(p\right)}\right)\underset{n\to \infty }{\leq }\frac{\gamma^{2}}{{\left(n-1\right)}^{2}}\left[\sum_{k=1}^{n-1}\frac{{\left[\ln\frac{k}{np}\right]}^{2}}{k}+w\underset{i<j}{\sum^{n-1}\sum^{n-1}}\left[\frac{\ln\left(\frac{i}{np}\right)\ln\left(\frac{j}{np}\right)}{\sqrt{i\cdot j}}\right]\right]; \end{array}$$

(22) $$\begin{array}{c} EFF{\left(\ln{\hat{Q}}_{New,\bar{H}}^{\left(p\right)}\right)}_{{}_{\ln{\hat{Q}}_{\bar{H}}^{\left(p\right)}\left(k\right)}}\underset{n\to \infty }{\geq }\frac{{\left(n-1\right)}^{2}\frac{{\left(\ln\left(\frac{k}{np}\right)\right)}^{2}}{k}}{\sum_{k=1}^{n-1}\frac{{\left[\ln\frac{k}{np}\right]}^{2}}{k}+\underset{i<j}{w\sum^{n-1}\sum^{n-1}}\left[\frac{\ln\left(\frac{i}{np}\right)\ln\left(\frac{j}{np}\right)}{\sqrt{i\cdot j}}\right]}>1,\\ \;\;\;\;\;for\;k=1,...,n-1, \end{array}$$

*where w is the weight, $w=\max_{i\neq j}\rho_{ij}^{+},$0≤w≤1,$\rho_{ij}^{+}=\left|\rho_{ij}^{}\right|,$$0\leq \rho_{ij}^{+}\leq 1;$$\rho_{ij}^{}$ is correlation coefficient of $\ln{\bar{Q}}_{\bar{H}}^{\left(p\right)}\left(i\right)$ and $\ln{\bar{Q}}_{\bar{H}}^{\left(p\right)}\left(j\right)$*

$$\rho_{ij}^{}=\frac{Cov\left[\ln{\bar{Q}}_{\bar{H}}^{\left(p\right)}\left(i\right),\ln{\bar{Q}}_{\bar{H}}^{\left(p\right)}\left(j\right)\right]}{\sqrt{\left(Var\left(\ln{\bar{Q}}_{\bar{H}}^{\left(p\right)}\left(i\right)\right)Var\left(\ln{\bar{Q}}_{\bar{H}}^{\left(p\right)}\left(j\right)\right)\right)}},\;i\neq j,i,\;j=1,...,n-1,\;-1\leq \rho_{ij}^{}\leq 1,\;\;and$$

$$EFF{\left(\ln{\hat{Q}}_{New,\bar{H}}^{\left(p\right)}\right)}_{\ln{\hat{Q}}_{\bar{H}}^{\left(p\right)}\left(k\right)}=\frac{Var\left(\ln{\bar{Q}}_{\bar{H}}^{\left(p\right)}\left(k\right)\right)}{Var\left(\ln{\hat{Q}}_{New,\bar{H}}^{\left(p\right)}\right)},\;\;where\;Var\left(\ln{\bar{Q}}_{\bar{H}}^{\left(p\right)}\left(k\right)\right)\underset{n\to \infty }{\approx }\gamma^{2}\frac{{\left(\ln\left(\frac{k}{np}\right)\right)}^{2}}{k}.$$

See Appendix A for the proof of Theorem 1.

### 3.3. The C.I. for The New Estimator ln ${\hat{Q}}_{New,\bar{H}}^{\left(p\right)}$

**Theorem** **2.** *Under conditions (C1) and (C2), a (1−α)100% confidence interval for $\ln VaR_{p}$ by using $\ln{\bar{Q}}_{New,\bar{H}}^{\left(p\right)}$ in (19) is given by*

(23) $$\left(LCL_{\ln{\hat{Q}}_{New,\bar{H}}^{\left(p\right)}}\left(k\right),\;UCL_{\ln{\hat{Q}}_{New,\bar{H}}^{\left(p\right)}}\left(k\right)\right)=\left(\ln{\hat{Q}}_{New,\bar{H}}^{\left(p\right)}-UCL_{\bar{H}}\left(k\right)b3,\;\ln{\hat{Q}}_{New,\bar{H}}^{\left(p\right)}+UCL_{\bar{H}}\left(k\right)b3\right)$$

where $z_{1-\alpha /2}$ *is the (1−α/2)th quantile of standard normal distribution, and*

$$b3=\frac{z_{1-\alpha /2}}{n-1}\sqrt{\sum_{k=1}^{n-1}\frac{{\left[\ln\left(\frac{k}{np}\right)\right]}^{2}}{k}+w\underset{i\neq j}{\sum^{n-1}\sum^{n-1}}\left[\frac{\ln\left(\frac{i}{np}\right)\ln\left(\frac{j}{np}\right)}{\sqrt{i\cdot j}}\right]},\;UCL_{\bar{H}}\left(k\right)=\frac{\bar{H}}{1-\;\frac{z_{1-\alpha /2}}{\sqrt{k}}}.$$

See Appendix A for the proof of Theorem 2.

**Remark** **1.** *Note that in the CI in (23), the main term $\ln{\hat{Q}}_{new,\bar{H}}^{\left(p\right)}$ does not depend on k, only the error terms $UCL_{\bar{H}}\left(k\right)b_{3}$ depends on k.*

**Remark** **2.** *In Section 4 Simulations and Section 5 Applications, we use the maximum weight w=1 in Formula (23), thus, we use maximum CI length for new proposed estimator ln${\hat{Q}}_{New,\bar{H}}^{\left(p\right)}$ comparing with existing methods. Even with maximum CI length. Section 4 and Section 5 show that the new estimator obtained confidence interval in (23) is still shorter than existing estimators obtained confidence intervals for most of k values.*

## 4. Simulations

### 4.1. Computer Simulations of Quantile Estimators

To verify that the new estimator $\ln{\hat{Q}}_{new,\bar{H}}^{\left(p\right)}$ has good properties, we use simulations and compare the new estimator to the existing estimators using the following statistics

1. The expected value E[·].
2. The root of mean squared errors RMSE[·].
3. The relative efficiencies REFF[·]

(24) $$REFF_{{\tilde{Q}}_{H\;\mathrm{or}\;\bar{H}}}=\sqrt{\frac{MSE\left[\ln q_{H}^{\left(p\right)}\left(k_{0}\right)\right]}{MSE\left[\ln{\tilde{Q}}_{{}_{H\;\mathrm{or}\;\bar{H}}}^{\left(p\right)}\left(k_{0}\right)\right]}}\;\mathrm{for}\;\tilde{Q}=Q\;\mathrm{or}\;\bar{Q},\;p=1/\left(2n\right),\;k_{0}\;\mathrm{is}\;\mathrm{defined}\;\mathrm{in}\;(15).$$

In this Section, we choose models of Fréchet (0.25), GPD (0.5), GPD (2) to compare with the simulation results of Gomes and Pestana (2007) [17]. We use four quantile estimators in Table 1 to run simulations. When $\left|\rho \right|\leq 1,$ estimators $\hat{\beta }$ and $\hat{\rho }$ in $\bar{H}$ use the tuning parameter τ=0, otherwise, use τ=1

(1) The Fréchet distribution (Fréchet, 1927) [22] has the c.d.f.
  (25) $$F\left(x\right)=\exp\left(-x^{-\frac{1}{\gamma }}\right),\;x>0,\;\;\gamma >0.$$
  An estimator of the *p*th ln-high quantile function is
  $$\ln Q_{\hat{\gamma }}^{\left(p\right)}=\ln x_{1-p,\hat{\gamma }}=-\hat{\gamma }\ln\left(\ln\left(\frac{1}{1-p}\right)\right),\;0<p<1,\;p\;\mathrm{is}\;\mathrm{very}\;\mathrm{small}.$$
(2) The generalized Pareto distribution (GPD) (de Zea Bermudeza and Kotz, 2010) [23] has the c.d.f.
  (26) $$F\left(x;\gamma \right)=1-{\left(1+\gamma x\right)}^{-\frac{1}{\gamma }},\;\;x\geq 0,\;\;\gamma \neq 0,$$
  for γ>0, an estimator of the *p*th ln-high quantile function is
  $$\ln Q_{\hat{\gamma }}^{\left(p\right)}=\ln VaR_{\hat{\gamma }}=\ln x_{1-p,\hat{\gamma }}=\ln\left(\frac{p^{-\hat{\gamma }}-1}{\hat{\gamma }}\right),\;0<p<1,\;p\;\mathrm{is}\;\mathrm{very}\;\mathrm{small}.$$

### 4.2. The Choice of α

As mentioned in Section 3, α is a key value to reduce the bias of the $\ln{\hat{Q}}_{New,\hat{;}}^{\left(p\right)}$ defined in (19). We developed an algorithm to estimate α based on the results of m− simulation runs:

**Step 1:** For a fixed sample size *n*, the $\alpha_{i}\left(n\right)$ in *i*th iteration, i=1,...,m, m=500, is the true solution of equation

$$\ln{\hat{Q}}_{New,{\bar{H}}_{i}}^{\left(p\right)}=\frac{1}{n-1}\sum_{k=1}^{n-1}\left[\ln X_{i,n-k+1:n}+{\bar{H}}_{i}\left(\ln\left(\frac{k}{np}\right)+\alpha C_{p}\left(k;\hat{\beta },\hat{\rho }\right)\right)\right]=\ln VaR_{p},i=1...,m,$$

then $\alpha \left(n\right)=\frac{1}{m}\sum_{i=1}^{m}\alpha_{i}\left(n\right).$ Note that α(n) depends on n. $\ln VaR_{p}$ is the true lnVaR value.

**Step 2:** Obtain estimator $\hat{\alpha }\left(n\right)$ based on the linear regression (LR) models where α is related to n. We collect data set $(\alpha_{j},n_{j}),$ j=1,...,l, with the sample size l.

(27) $$\hat{\alpha }\left(n\right)=\left\{\begin{array}{ll} \hat{\rho }(n), & \left|\rho (n)\right|<1,\;\mathrm{for}\;GPD(0.5);\\ {1.7488-0.0002n+2.9693X}_{1}{+2.6604X}_{2}, & \left|\rho (n)\right|\geq 1,\;\\ \; & \mathrm{for}\;\mathrm{Fréchet}\;(0.25),X_{1}=\hat{\beta }{(n);X}_{2}=\hat{\rho }(n);\;\\ \; & \mathrm{for}\;\mathrm{GPD}(2),X_{1}=\hat{\rho }{(n);X}_{2}=\hat{\beta }(n).\; \end{array}\right.$$

Note that the estimate $\hat{\alpha }\left(n\right)$ in (27) depends the parameters of the models and LR relationship with sample size *n*.

**Remark** **3.** *If we assume $\alpha_{j}$ in $(\alpha_{j},n_{j}),$j=1,...,l, is normally distributed, based on (Bickel and Doksum, 2015, pp. 286–388) [24], then $\hat{\alpha }\left(n\right)$ is a maximum likelihood estimator (MLE) and has an asymptotic normal distribution. Since the estimator $\hat{\alpha }\left(n\right)$ only depands to n not related to the order statistics, it will not affect the asymptotic proprties of the proposed estimator $\ln{\hat{Q}}_{New,\hat{\gamma }}^{\left(p\right)}$ in* (19).

### 4.3. Simulation of $Fr\overset{´}{e}chet$ (0.25). GPD (0.5) and GPD (2)

Table 2, Table 3 and Table 4 list the results of simulations under the Fréchet (0.25), GPD (0.5) and GPD (2), where N=500 iterations for sample size n=500,1000,2000,5000 and p=1/(2n). With $\hat{\alpha }\left(n\right)$ in (27), we compare mean values, mean squared errors (MSE) and REFF of the four lnVaR estimators in Table 1, at optimal level $k=k_{0}$ based on (15) Note that the new estimator $\ln{\hat{Q}}_{New,\hat{\gamma }}^{\left(p\right)}$ has the highest REFF values among the four estimators which are in bold in all three models. The simulation MSE of $\ln{\hat{Q}}_{New,\hat{\gamma }}^{\left(p\right)}$ is defined as

$$MSE\left(\ln{\hat{Q}}_{New,\hat{\gamma }}^{\left(p\right)}\right)=\frac{1}{N}\sum_{i=1}^{N}{\left(\ln{\hat{Q}}_{New,\hat{\gamma },i}^{\left(p\right)}-\ln VaR_{p}\right)}^{2},$$

where $\ln{\hat{Q}}_{New,\hat{\gamma },i}^{\left(p\right)}$ is the $\ln{\hat{Q}}_{New,\hat{\gamma }}^{\left(p\right)}$ in the *i*th iteration, i=1,...,N. So do for other ln-quatile estimators.

**Figure 1:**
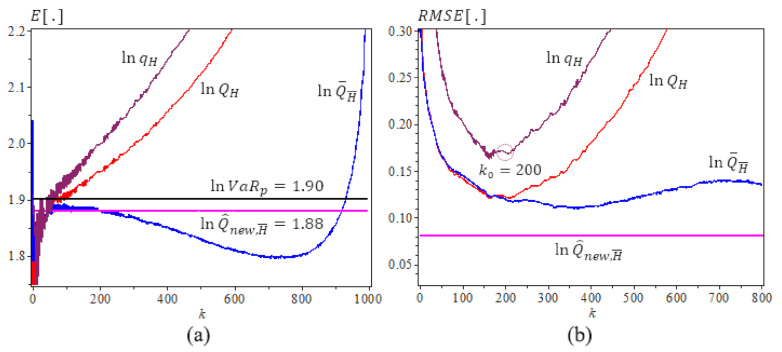
Underlying *Fréchet* (0.25), ρ=−1, β=0.5. N=500, n=1000. (**a**) The means of ln-quantile estimators with the true $\ln VaR_{0.0005}\approx 1.9$ ($\ln{\hat{Q}}_{new,\bar{H}}^{0.0005}\approx 1.88$). (**b**) The RMSE of Ln-quantile estimation, p=0.0005, $\hat{\alpha }=1.14.$

**Figure 2:**
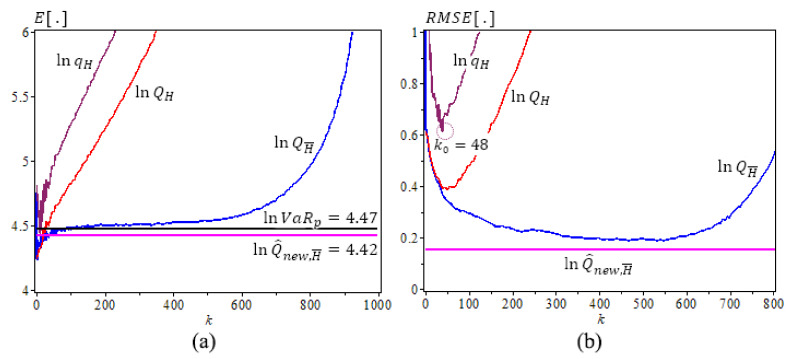
Underlying GPD (0.5), ρ=−0.5, β=1, N=500, n=1000. (**a**) The means of ln-quantile estimators with the true $\ln VaR_{0.0005}\approx 4.47$ $(\ln{\hat{Q}}_{new,\bar{H}}^{0.0005}\approx 4.42).$ (**b**) The RMSE of Ln-quantile estimation, p=0.0005, $\hat{\alpha }=-0.7482$.

**Figure 3:**
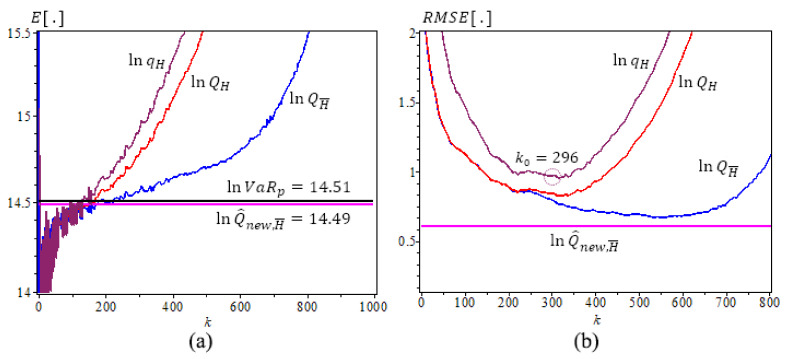
Underlying GPD (2), ρ=−2, β=1, N=500, n=1000. (**a**) The means of ln-quantile estimators with the true $\ln VaR_{0.0005}\approx 14.51$ ($\ln{\hat{Q}}_{new,\bar{H}}^{0.0005}\approx 14.49$). (**b**) The RMSE of ln-quantile estimators, p=0.0005, $\hat{\alpha }=-2.8417.$

Figure 1, Figure 2 and Figure 3 are based on Table 2, Table 3 and Table 4 results, Figure 1 is for Fréchet (0.25), we use N=500 iterations, sample size n=1000, γ=0.25, ρ=−1, β=0.5, p=1/2n. The new estimator $\ln{\hat{Q}}_{New,\hat{\gamma }}^{\left(p\right)}$ has the best performance with the least bias and *RMSE*. It does not change as *k* varies. Figure 2 and Figure 3 are for GPD(0.5) and GPD(2), N=500 iterations, sample size n=1000, γ=0.5 and 2, ρ=−γ, β=1, p=1/2n. We note that the new estimator $\ln{\hat{Q}}_{new,\bar{H}}$ is the best estimator as well, with the least bias, consistency as *k* varies, and the smallest RMSE. Note that $\ln{\hat{Q}}_{New,\hat{\gamma }}^{\left(p\right)}$ values are very close to the true lnVaR${}_{p}$ values.

### 4.4. Simulations of Confidence Intervals

By Gomes and Pestana (2007) [17], the 95% confidence interval of the true tail index using *H* is

(28) $$\left(LCL_{H}\left(k\right),UCL_{H}\left(k\right)\right)=\left(\frac{H(k)}{1+\frac{\beta {\left(n/k\right)}^{\rho }}{1-\rho }+\frac{1.96}{\sqrt{k}}},\frac{H(k)}{1+\frac{\beta {\left(n/k\right)}^{\rho }}{1-\rho }-\frac{1.96}{\sqrt{k}}}\right)$$

and the 95% confidence interval of the true tail index using $\bar{H}$ is

(29) $$\left(LCL\bar{{}_{H}}\left(k\right),UCL\bar{{}_{H}}\left(k\right)\right)=\left(\frac{\bar{H}\left(k\right)}{1+\frac{1.96}{\sqrt{k}}},\frac{\bar{H}\left(k\right)}{1-\frac{1.96}{\sqrt{k}}}\right)$$

Next, we compute the confidence intervals for the true ln-quantile by using the quantile estimators. We only use three out of four quantile estimators in Table 1, except $\ln q_{H}$ which has the worst result. Therefore, we compare CIs only using $\ln Q_{H}$, $\ln{\tilde{Q}}_{\bar{H}}$ and $\ln{\hat{Q}}_{new,\bar{H}}$ in (30), (31) and (23). Thus

(1) The 95% confidence interval for the true $\ln VaR_{p}$ using $\ln Q_{H}$ is
  (30) $$\begin{array}{ccc} LCL_{\ln Q_{H}}\left(k\right) & = & \min\ln Q_{H}\left(k\right)-LCL_{H}\left(k\right)\ln\left(\frac{k}{np}\right)b_{2},\ln Q_{H}\left(k\right)-UCL_{H}\left(k\right)\ln\left(\frac{k}{np}\right)b_{2};\\ UCL_{\ln Q_{H}}\left(k\right) & = & \max\ln Q_{H}\left(k\right)+LCL_{H}\left(k\right)\ln\left(\frac{k}{np}\right)b_{1},\ln Q_{H}\left(k\right)+UCL_{H}\left(k\right)\ln\left(\frac{k}{np}\right)b_{1}. \end{array}$$
  where $LCL_{H}\left(k\right)$, $UCL_{H}\left(k\right)$ is given in (28), and
  $$b_{1}=\frac{1.96}{\sqrt{k}}-\frac{\beta {\left(n/k\right)}^{\rho }}{1-\rho },\;b_{2}=\frac{1.96}{\sqrt{k}}+\frac{\beta {\left(n/k\right)}^{\rho }}{1-\rho }.$$
(2) The 95% confidence interval for the true $\ln VaR_{p}$ using $\ln{\tilde{Q}}_{\bar{H}}$ is
  (31) $$\begin{array}{ccc} LCL_{\ln\tilde{Q}\bar{{}_{H}}}\left(k\right) & = & \ln\tilde{Q}\bar{{}_{H}}-UCL_{\bar{H}}\left(k\right)\ln\left(\frac{k}{np}\right)\frac{1.96}{\sqrt{k}},\\ UCL_{\ln\tilde{Q}\bar{{}_{H}}}\left(k\right) & = & \ln\tilde{Q}\bar{{}_{H}}+UCL_{\bar{H}}\left(k\right)\ln\left(\frac{k}{np}\right)\frac{1.96}{\sqrt{k}}; \end{array}$$
  where $LCL_{\bar{H}}\left(k\right)$, $UCL_{\bar{H}}\left(k\right)$ is given in (29), and
(3) The 95% confidence interval for the true $\ln VaR_{p}$ using $\ln{\hat{Q}}_{new,\bar{H}}$ is given in (24).

To compare new proposed CI in (23) to CIs in (30) and *(31)*, we use evaluate the length and probability coverage of the CIs.

The length of CI is given as

$$length\;\mathrm{of}\;\mathrm{CI}=UCL_{\mathrm{quantile}\;\mathrm{estimator}}\;-LCL_{\mathrm{quantile}\;\mathrm{estimator}.}\;$$

and the efficiency of the length of 95% CI is given as

(32) $$EFF_{length}=\frac{C.I.\;length\;of\;\ln Q_{H}\;\mathrm{at}\;k_{0}^{Q_{H}}}{C.I.\;length\;of\;\ln\bar{Q}\bar{{}_{H}}\;\mathrm{or}\;\ln{\hat{Q}}_{new,\bar{H}}\;\mathrm{at}\;k_{01}}.$$

Also, the confidence interval is more efficient when it has a higher coverage of the true value under the simulations, where the probability coverage of 95% CI is defined as

$$P.C.=\frac{\mathrm{number}\;\mathrm{of}\;95\%\;CI’s\;\mathrm{contains}\;\mathrm{the}\;\mathrm{true}\;\mathrm{value}}{\mathrm{number}\;\mathrm{of}\;95\%\;CI’s\;\mathrm{simulated}\;\mathrm{in}\;\mathrm{total}}*100\%.$$

and the efficiency of the probability coverage of 95% CI is given as

(33) $$EFF_{P.C.}=\frac{|P.C._{\ln Q_{H}}-95\%|}{\left|\left(P.C._{\ln\bar{Q}\bar{{}_{H}}}\;\mathrm{or}\;P.C._{\ln{\hat{Q}}_{new,\bar{H}}}\right)-95\%\right|}.$$

when $EFF_{P.C.}$ is bigger means it is more efficient.

**Figure 4:**
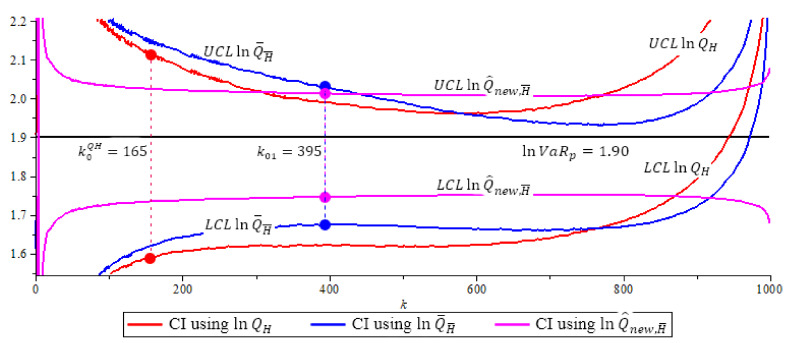
$Fr\overset{´}{e}chet$ (0.25) model, 95% confidence interval of quantile estimators, N=500, n=1000, p=0.0005, β=0.5, ρ=−1, α(1000)=LR=1.14, $k_{0}^{Q_{H}}=165,$ $k_{01}=395.$ Note that $\ln{\hat{Q}}_{new,\bar{H}}$ (purple) has shortest CI with length 0.2668. (The solid circles “•” in the plot are the values of the quantile estimators at their optimal *k* level).

**Figure 5:**
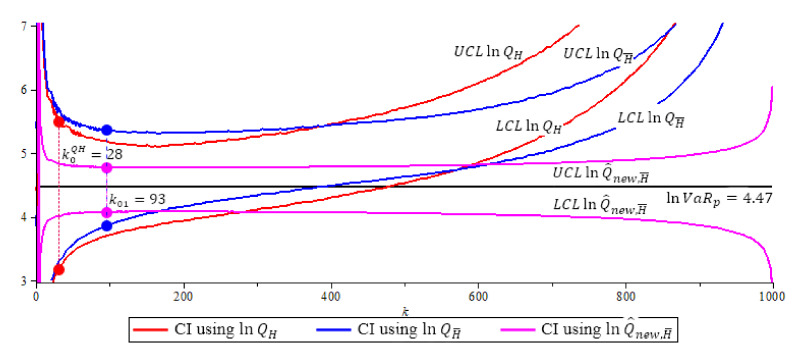
The GPD (0.5) model, 95% confidence interval of quantile estimators, N=500, n=1000, p=0.0005, β=1,ρ=−0.5, $\hat{\alpha }\left(1000\right)=\hat{\rho }=-0.7482,$ $k_{0}^{Q_{H}}=28,$ $k_{01}=93.$ Note that $\ln{\hat{Q}}_{new,\bar{H}}$ (purple) has shortest CI with length 0.7094. (The solid circles “•” in the plot are the values of the quantile estimators at their optimal *k* level).

**Figure 6:**
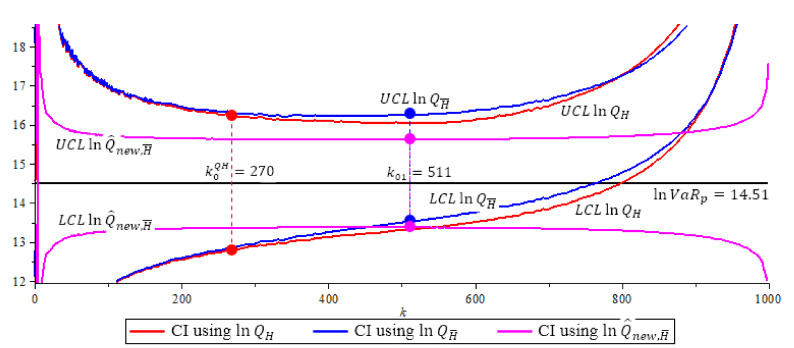
The GPD (2) model, 95% confidence interval of quantile estimators N=500,n=1000, p=0.0005, β=1,ρ=−2, $\hat{\alpha }\left(1000\right)=LR=-2.8417,$ ${\hat{k}}_{0}=75,$ ${\hat{k}}_{0}^{Q_{H}}=80,$ ${\hat{k}}_{01}=70.$ Note that $\ln{\hat{Q}}_{new,\bar{H}}$ (purple) has shortest CI with length 2.2511. (The solid circles “•” in the plot are the values of the quantile estimators at their optimal *k* level).

Figure 4, Figure 5 and Figure 6 show the 95% confidence interval of the three ln-quantile estimators under Fréchet (0.25), GPD (0.5 and 2) with p=0.0005. We compare the size of each confidence interval at their optimal *k* level, and the probability coverage of each confidence interval at their optimal *k* level. Recall, the optimal *k* level for $\ln Q_{H}$ is at $k_{0}^{Q_{H}}$ based in (16), the optimal *k* level for $\ln\tilde{Q}\bar{{}_{H}}$ and $\ln{\hat{Q}}_{new,\bar{H}}$ is at $k_{01}$ based in (15).

Table 5 compare the efficiencies of 95% CI of the three quantile estimators under $Fr\overset{´}{e}chet$ (0.25), GPD (0.5 and 2). The efficiency of 95% CI can be compared by the length of CI and the probability coverage of CI, denoted by $EFF_{length}$ and $EFF_{P.C.}$.

In this section, we compared the new quantile estimator $\ln{\hat{Q}}_{new,\bar{H}}^{\left(p\right)}$ in (19) with the existing methods. $\ln{\hat{Q}}_{new,\bar{H}}^{\left(p\right)}$ has the least bias, the smallest RMSE, and not depends on *k* too much. It also has the smallest length and the highest probability coverage in 95% confidence interval in most cases. The simulation results verify that $\ln{\hat{Q}}_{new,\bar{H}}$ is the best quantile estimator among all three methods. Next section, we apply the new estimator $\ln{\hat{Q}}_{new,\bar{H}}^{\left(p\right)}$ to real world examples.

## 5. Applications

We will study two real-world examples in this Section. We are interested in the population that is above the threshold for each example. The goal is to estimate the (1−p)th high quantiles of the example, where 0<p<1 is a very small. We use the four quantile estimators in table 1 $\ln q_{H},$ $\ln Q_{H},$ $\ln Q_{\bar{H}}$ and $\ln{\hat{Q}}_{new,\hat{\gamma }}$ in (21), and compare their performances.

- Procedure:
  Step 1: Choose and collect data of examples of real life extreme events.
  Step 2: Run Goodness-of-Fit tests to check if data is heavy distributed.
  Step 3: Estimate the high quantiles and construct the confidence intervals by using the new method and the existing methods.
  Step 4: Estimate the high quantiles and construct the confidence intervals by using the new method and the existing methods.
- Estimators
  1. Two tail index estimators H(k) in (5) and $\bar{H}\left(k\right)$ in (9).
  2. Four quantile estimators (6), (7), (12) and (19) are in Table 1.
  3. We use $\hat{\alpha }\left(n\right)$ in (27) for the new estimator $\ln{\hat{Q}}_{new,\bar{H}}^{\left(p\right)}$ in (19) for the GPD model.

**Remark** **4.** *In applications, the GPD is used as a tail approximation to the population distribution from which a sample of excesses x−μ above some suitably high threshold μ are observed. The GPD is parameterized by location, scale and shape parameters μ,λ>0 and γ, and can equivalently be specified in terms of threshold excesses x−μ or, as here, exceedances x>μ, as three parameters (γ,μ,λ)* GPD *in (34) (de Zea Bermudeza and Kotz, 2010) [23],*

(34) $$H_{\gamma }\left(x\right)=1-{\left(1+\gamma \frac{x-\mu }{\lambda }\right)}^{-\frac{1}{\gamma }},\;0<\mu <x<{\left(0\vee \left(-\gamma \right)\right)}^{-1},\;\lambda >0,$$

*Traditionally, the threshold was chosen before fitting, giving the so-called fixed threshold approach (Pickands, 1975 [25], Balkema and de Haan, 1974 [26]). It is common for practitioners to assume a constant quantile level, determined by some assessment of fit across all or a subset of the datasets (Scarrott and McDonald, 2012, p.36 [27]). In our application, the threshold is pre-determined by physical considerations, that is, number of type A flu viruses detected weekly in Canada above the average in flu season, and the counts of gamma ray released from significant solar flares (M and X rated) during the Sun’s active years. Although it is possible to make some arbitrary definition of the choice of the threshold, it is preferable not to become involved with such delicate question. The application of the proposed method is presented in both examples for illustrative purpose.*

### 5.1. Flu in Canada Example

According to the WHO (World Health Organization, 2020 [28]), seasonal influenza is a common infection of the airways and lungs that can spread easily among humans. There are 37 million people in Canada, and flu season usually runs from November to April. Most people recover from the flu in about a week. However, influenza may be associated with serious complications such as pneumonia, especially in infants, the elderly and those with underlying medical conditions like diabetes, anemia, cancer, and immune suppression. On average, the flu and its complications send about 12,200 Canadians to the hospital every year, and around 3500 Canadians die. There are 3 types of flu viruses, A, B and C. Type A flu virus is the most harmful, and it is constantly changing and is generally responsible for the large flu epidemics. The 1918 Spanish Flu, 1957 Asian Flu, 1968 Hong Kong Flu, 2009 Swine flu, and the most recent 2014 H5N1 Bird Flu are all type A flu. In this paper, we study type A viruses in Canada.

We collected the number of the type A flu viruses detected weekly in Canada, from 1 January 1997 to 31 December 2019, resulting in a sample size of $n^{*}=$ 994 weeks. According to the WHO, the average number of type A flu viruses detested per week in the flu season, November to April, is 953, for the past 10 years. We set 953 viruses/week as the threshold, which reduced our sample size to n= 111 weeks. Full data-set is available at http://apps.who.int/influenza/gisrs_laboratory/flunet/en.

**Figure 7:**
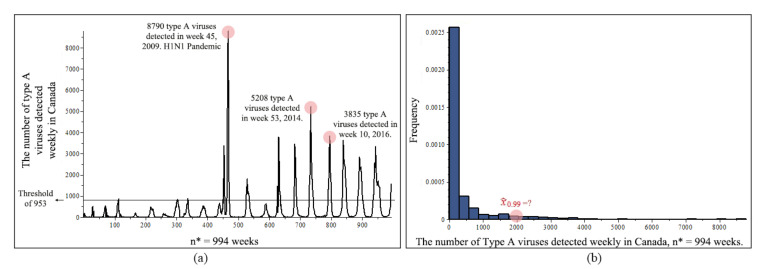
Flu original data from 1 January 1997 to December 31 2019, $n^{*}=$ 994 weeks, (**a**) Flu chart of type A flu viruses detected in Canada, and n= 111 weeks remaining after the threshold, of average 953 flu viruses. (**b**) Histogram of the number of type A flu viruses detected in Canada.

Figure 7a shows a Flu chart in $n^{*}=$ 994 weeks of type A flu viruses detected in Canada, and n= 111 weeks remaining after the threshold, of average 953 flu viruses. For each flu incubation period, a flu virus can last from one up to few weeks, that is why some arches are narrow and some arches are more bell shaped in this figure. The top three weeks are circled in the plot. Figure 7b shows a histogram of $n^{*}=$ 994 weeks data. We are interested in the 99% quantile, $x_{0.99}$, such that 99% chance that the viruses detected in a given week would be less than this value, or equivalently, with a 1% possibility, the number of flu viruses detested in a given week would be in excess of this value. This information is useful for monitoring and studying the virus, also is helpful for medical organizations that deal with disease control and prevention, pharmaceutical availability, and hospital resource readiness, especially during a serious flu outbreak. ${\hat{x}}_{0.99}$ is approximately located in the plot. In this paper, we propose a new estimate high quantiles method, and compare it with existing methods.

Our interest is to find the 5%VaR and 1% VaR of the number of type A flu viruses detested in a week, and their 95% confidence intervals.

#### 5.1.1. Goodness-of-Fit Test

**Figure 8:**
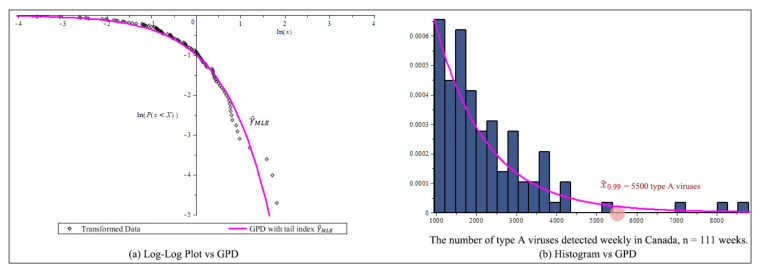
After threshold 953 flu viruses, Flu transformation data, n=111, (**a**) Log-log plot of flu in Canada example. (**b**) Estimate GPD curve and the 99% high quantile and histogram of the distribution of type A flu viruses detested weekly.

Through data transformation $Y_{i}=\frac{X_{i}-\mu }{\lambda }$, i=1,...,n, n=111. Take μ=953 as the threshold, the maximum likelihood estimators (MLE) are ${\hat{\lambda }}_{MLE}=1275.97287$ and ${\hat{\gamma }}_{MLE}=0.01345.$ Figure 8a is the log-log plot of GPD curve with the horizontal axis ln(x) against the vertical axis ln(P{x<X}). Visually the transformed data fit the one parameter GPD in (26) the bestusing ${\hat{\gamma }}_{MLE}$ (red curve). Figure 8b shows the GPD density curve (red curve) fits the histogram very well.

Beside visual view of Figure 8, we also carry on the three goodness-of-fit tests: the Kolmogorov-Smirnov (*K-S*) test (Kolmogorov, 1933 [29]), Anderson-Darling (*A-D*) test, and Cramér von Mises (*C-v-M*) test (Anderson-Darling, 1952 [30]). All three tests are based on the maximum vertical distance between the empirical distribution function and the observations, and the parent distribution function is the GPD.

The Hypothesis for all three tests is

$$\begin{array}{ccc} H_{0} & : & \;F\left(x\right)=F^{*}\left(x\right),\;\mathrm{for}\;\mathrm{all}\;\mathrm{values}\;\mathrm{of}\;x\\ H_{1} & : & \;F\left(x\right)\neq F^{*}\left(x\right),\;\mathrm{for}\;\mathrm{at}\;\mathrm{least}\;\mathrm{one}\;\mathrm{value}\;\mathrm{of}\;x \end{array}$$

F(x) is the true but unknown distribution of the sample. $F^{*}\left(x\right)$ is the theoretical distribution, in our project, the parent distribution, GPD. $S_{n}\left(x\right)$ is the empirical distribution and step function of the sample. It is defined as

$$S_{n}\left(x\right)=\frac{1}{n}\overset{n}{\sum_{i=1}}I_{\left(-\infty ,x\right]}\left(X_{i}\right),\;\mathrm{where}\;I_{A}=\left\{\begin{array}{cc} 1, & \mathrm{if}\;x\in A;\\ 0 & \mathrm{if}\;x\notin A. \end{array}\right.$$

where −∞<x<∞, $0\leq S_{n}\left(x\right)\leq 1.$

The test statistics under $H_{0}$ of K−S test is

(35) $$T=\underset{x}{\sup}\left|F^{*}\left(x\right)-S_{n}\left(x\right)\right|.$$

Based on Table 6 goodness of fit tests’ results, we set the GPD model for the flu in Canada data. We define the absolute errors (AE) in (34) and integrated errors (IE) in (35) as

(36) $$IE=\frac{1}{X_{n:n}-X_{n-r+1:n}}{\left(\int_{X_{n-r+1:n}}^{X_{n:n}}{\left[S_{n}\left(x\right)-F^{*}\left(x\right)\right]}^{2}dx\right)}^{1/2}.$$

For both AE and IE, we use 3 different *r* values by letting $r=\frac{n}{10}th,$ $r=\frac{n}{2}th$, and r=nth top statistics. Table 7 lists the *AE* and *IE* errors which are very small.

Next, we estimate the high quantiles and their confidence interval for this example.

#### 5.1.2. Compare Four Estimation Methods

We use the four estimators in Table 1: $\ln q_{H},$ $\ln Q_{H},$ $\ln Q_{\bar{H}}$, and the new estimator $\ln{\hat{Q}}_{new,\bar{H}}$.

We use ${\hat{\rho }}_{\tau }\left(k\right)$ in (10), and ${\hat{\beta }}_{{\hat{\rho }}_{0}}\left(k\right)$ in (11). To decide if the tuning parameter τ=0 or 1, consider ${\left\{{\hat{\rho }}_{\tau }\left(k\right)\right\}}_{k\in k}$, for $k\in k=(\left[n^{0.995}\right],\left[n^{0.999}\right])$, and compute their median $x_{\tau }$, then

$$\tau =\underset{k}{\arg\min}\sum_{k\in k}{\left({\hat{\rho }}_{\tau }\left(k\right)-x_{\tau }\right)}^{2}.$$

**Figure 9:**
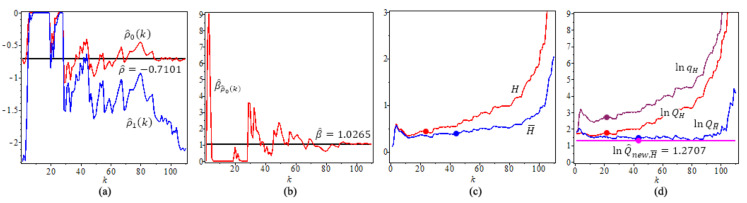
For flu in the Canadian data, n=111, (**a**) Estimates of the second-order parameter $\hat{\rho }$ and ${\hat{\rho }}_{\tau }\left(k\right)$, τ=0; (**b**) Estimates $\hat{\beta }$ and ${\hat{\beta }}_{{\hat{\rho }}_{0}}\left(k\right).$ (**c**) Tail index estimators, *H*, $\bar{H};$ (**d**) ln-quantile estimators, p=0.01. The solid circles “•” in the plot are the values of the quantile estimators at their optimal *k* level.

With n=111, we get k∈k=(108,110) and $x_{\tau }=109$, then $\sum_{k\in k}{\left({\hat{\rho }}_{0}\left(k\right)-x_{\tau }\right)}^{2}\approx 36116<\sum_{k\in k}{\left({\hat{\rho }}_{1}\left(k\right)-x_{\tau }\right)}^{2}\approx 37033$, conclude that τ=0, thus we have ${\hat{\rho }}_{0}\left(k_{1}\right)=-0.7101$ and ${\hat{\beta }}_{{\hat{\rho }}_{0}}\left(k_{1}\right)=1.026571$, where $k_{1}$ is the optimal *k* value. Figure 9 shows the results.

Figure 9a shows estimates of the second-order parameters ρ through $\hat{\rho }$ and ${\hat{\rho }}_{\tau }\left(k\right)$, τ=0; Figure 9b shows Estimates $\hat{\beta }$ and ${\hat{\beta }}_{{\hat{\rho }}_{0}}\left(k\right).$ Figure 9c shows the two estimated tail index, *H*, $\bar{H},$ H=0.4379 at its optimal level using ${\hat{k}}_{0}=21$ based on (15) and $\bar{H}=0.3736$ at its optimal level using ${\hat{k}}_{01}=42$ based on (17). Figure 9d shows four quantile estimators of flu in Canada example, with p=0.01. The full circles “•” in the plot are the values of the quantile estimators at their optimal *k* level. We note that $\ln{\hat{Q}}_{new,\bar{H}}^{\left(p\right)}$ has a constant value, which does not depend on k.

**Figure 10:**
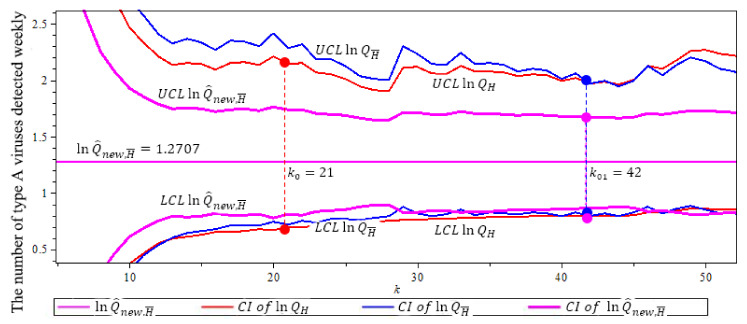
95% confidence interval of three ln-quantile estimators after the threshold 953 for the flu in Canada example. n=111, p=0.01. Note that $\ln{\hat{Q}}_{new,\bar{H}}$ (purple) has shortest CI with length 0.7966. (The solid circles “•” in the plot are the values of the quantile estimators at their optimal *k* level).

Figure 10 compares the confidence intervals of three quantile estimators in (7), (12) and (19). This figure shows that the new quantile estimator $\ln{\hat{Q}}_{new,\bar{H}}^{\left(p\right)}$ has the smallest confidence interval with length 0.7966, where we use $\hat{\alpha }=\hat{\rho }=-0.7101.$ (The solid circles “•” in the plot are the values of the quantile estimators at their optimal *k* level).

In Table 8, we compare the four ln-quantile estimators and their mean, median, $VaR_{0.05}$ and $VaR_{0.01}.$ Table 9 compares the size of confidence intervals at $\ln VaR_{0.01}$ and $VaR_{0.01}$ of the three quantile estimators.

In Table 9, we compared $Q_{H}$, $Q_{\bar{H}}$ and $Q_{new,\bar{H}},$ the $Q_{new,\bar{H}}$ has the shortest confidence interval with the highest efficiency of 2.2462.

#### 5.1.3. Summary

Based on Figure 10 and Table 9, we conclude that the new estimator $\ln{\hat{Q}}_{new,\bar{H}}$ in (19) is the best estimator for Flu in Canada example. We can predict that at $VaR_{0.01}$, we expect 5500 type A flu viruses during a flu outbreak after threshold 953/week. This is shown in Figure 8b.

### 5.2. Gamma Ray of Solar Flare Example

Gamma ray has the most penetrating power among all the radiations. The burst of gamma rays are thought to be, due to the collapse of stars called hypernovas, the most powerful events so far discovered in the cosmos. The measurement of gamma rays are in counts, and it is the number of atoms in a given quantity of radioactive material that are detected by an instrument to have decayed. We have collected gamma ray data from solar flares, from November 2008 to September 2020, from NASA (National Aeronautics and Space Administration, 2020 [31]). Full data-set is available at http://hesperia.gsfc.nasa.gov/fermi/gbm/qlook/fermi_gbm_flare_list.txt.

**Figure 11:**
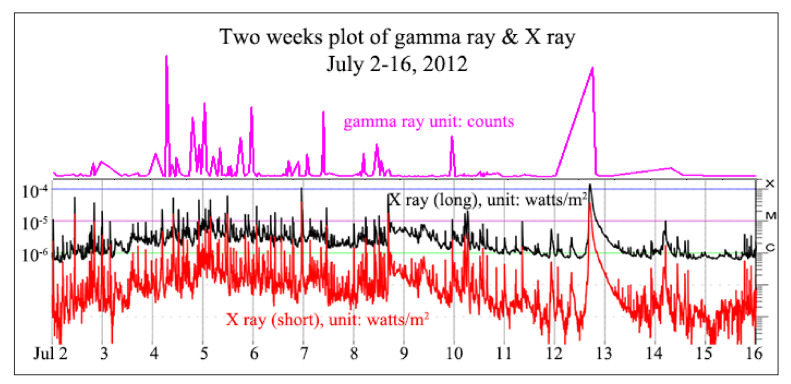
Two weeks plot of gamma ray & X ray from July 2 to 16, 2012.

The solar flare travels hundreds of miles per second, and can reach the Earth within hours. It can disrupt communication navigational equipment, damage satellites, and even cause blackouts by damaging power plants. In 1989, a strong solar storm knocked out the power grid in Québec, Canada, causing 6 million people to lose power for more than 9 hours, and it cost millions of dollars to repair. It can bring additional radiation around the north and south poles, a risk that forces airlines to reroute flights. The Fermi Gamma-ray Space Telescope was launched in late 2008 to explore high-energy phenomena in the Universe. It is worth noting that more than one trigger may have occurred during the flare, the one nearest the peak of the flare is listed, resulting in a sample size of 5128. Solar flares are classified as A, B, C, M or X according to the peak flux (in watts per square meter, W/m^2^) of 1 to 8 angstrom (The angstrom is a unit of length equal to 1/10,000,000,000 (one ten-billionth) of a meter.) X-rays near the Earth, as measured on the GOES spacecraft. Gamma ray activity is correlated with the X ray activity, as shown in Figure 11 (NOAA, 2020 [32]. When the amount of gamma ray released is over 5 million counts, it usually corresponds to an X rated flare or significant M rated flares.

**Figure 12:**
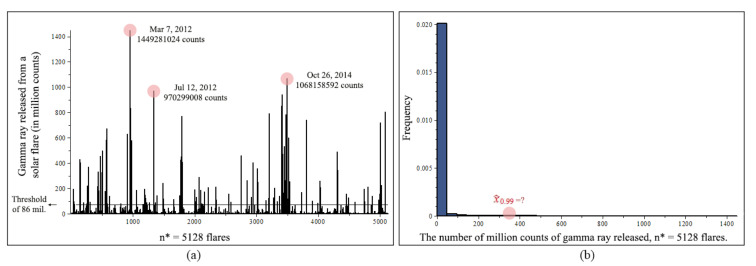
Gamma ray original data from November 2008 to April 2017, $n^{*}=5182,$ (**a**) Gamma ray released V.S solar flare occurred. After the threshold of 86 million counts, n=104 flares remaining. (**b**) Histogram of gamma ray released from solar flares.

Figure 12a shows a Gamma ray chart of $n^{*}=$ 5128 flares, and n= 104 flares remaining after the threshold of 86 million counts. The most powerful gamma ray was released in March 7, 2012 with nearly 1.5 billion counts, the sun was brightened by 1000 times, and became the brightest object in the gamma ray sky. The top three events are circled in the chart. Figure 12b shows a histogram of $n^{*}=$ 5128 flares. We are interested in the 99% quantile, $x_{0.99}$, such that 99% gamma ray released from solar flares are under this value, or equivalently, with a 1% possibility, the amount of gamma ray a solar flare releases would be in excess of this value. During the spring and fall, the satellites that are used to detect solar flares experience eclipses, in which the Earth or the Moon blocks between the satellites and the Sun for a short period every day. Eclipse season lasts for about 45 to 60 days and ranges from minutes to just over an hour. The quantile estimation would provide useful predictions for these times. ${\hat{x}}_{0.99}$ is approximately located in the plot since we do not know this value yet.

We chose the threshold as the mean of the data from the peak period. The solar cycle is every 11.6 years, and the sun’s activity peaked from 2011 to 2014. In Figure 12a we can see that the top 3 flares, in fact, almost 90% of the top 100 flares, are from the 2011 to 2014 time period. Taking the average of all the X rated and significant M rated flares from this peak period, we obtained a mean of 86 million counts, resulting in a remaining sample size of n=104.

For the Gamma ray of solar flare example, our goal is to find out the high quantiles, specifically, the 5% VaR and 1% VaR of the amount of gamma ray a solar flare would release, and their 95% confidence intervals.

#### 5.2.1. Gooness-of-Fit Tests

**Figure 13:**
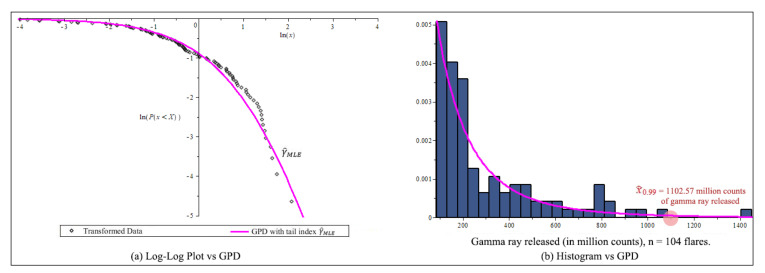
After threshold 86 millions count, transformation data, n=104, (**a**) Log-log plot of gamma ray from solar flare example. (**b**) The Estimate GPD and the 99% high quantile of the distribution of gamma ray released by solar flare.

Similar as Flu in Canada Example, we set μ=86million, and obtain ${\hat{\lambda }}_{MLE}=171.0708592$, and ${\hat{\gamma }}_{MLE}=0.2580384847.$ Figure 13a is a log-log plot of gamma ray data under GPD model, with the horizontal axis ln(x) against the vertical axis ln(P{x<X}). Figure 13b shows the histogram fits the GPD model.

Next, we will perform three goodness-of-fit tests: Kolmogorov-Smirnov test, the Anderson-Darling test and the Cramér-von-Mises test. The results listed in Table 10, the data fits the GPD with ${\hat{\gamma }}_{MLE}$ the best, nearly 59%.

In Table 11, all the errors are less than 0.07 for *AE*, and less than 0.01 for IE.

Next, we can compare the four high quantile estimators and their confidence intervals of this example.

#### 5.2.2. Compare Four Estimation Methods

Similar as Example 1, we use the four quantile estimators in Table 1: $\ln q_{H}$, $\ln Q_{H}$, $\ln Q_{\bar{H}}$, and the $\ln{\hat{Q}}_{new,\bar{H}}$.

**Figure 14:**
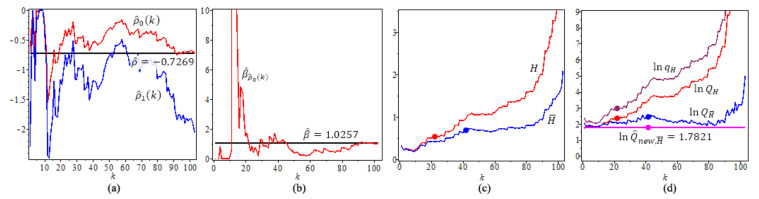
For gamma ray of solar flare example, n=104, (**a**) Estimates of the second-order parameters $\hat{\rho }$ and ${\hat{\rho }}_{\tau }\left(k\right)$, τ=0, (**b**) Estimates $\hat{\beta }$ and ${\hat{\beta }}_{{\hat{\rho }}_{0}}\left(k\right).$ (**c**) Tail index estimators, *H*, $\bar{H}$. (**d**) ln-quantile estimators, p=0.01. The solid circles “•” in the plot are the values of the quantile estimators at their optimal *k* level.

We use ${\hat{\rho }}_{\tau }\left(k\right)$ and ${\hat{\beta }}_{{\hat{\rho }}_{0}}\left(k\right),$ and τ=0, thus we have ${\hat{\rho }}_{0}\left(k1\right)=-0.7269$ and ${\hat{\beta }}_{{\hat{\rho }}_{0}}\left(k_{1}\right)=1.0257$, where $k_{1}$ is the optimal *k* value for the second-order parameters. The results are in Figure 14.

Figure 14a shows the estimates of the second-order parameters $\hat{\rho }$ and ${\hat{\rho }}_{\tau }\left(k\right)$, τ=0. Figure 14b shows $\hat{\beta }$ and ${\hat{\beta }}_{{\hat{\rho }}_{0}}\left(k\right).$ Figure 14c shows the two different tail index estimators, *H*, $\bar{H}$. We have H=0.5324 at its optimal level with ${\hat{k}}_{0}=21$, $\bar{H}=0.6517$ at its optimal level with ${\hat{k}}_{01}=41.$ Figure 14d shows all four quantile estimators of gamma ray example, with p=0.01. We note that $\ln{\hat{Q}}_{new,\bar{H}}$ has a constant value which does not depend on *k*.

**Figure 15:**
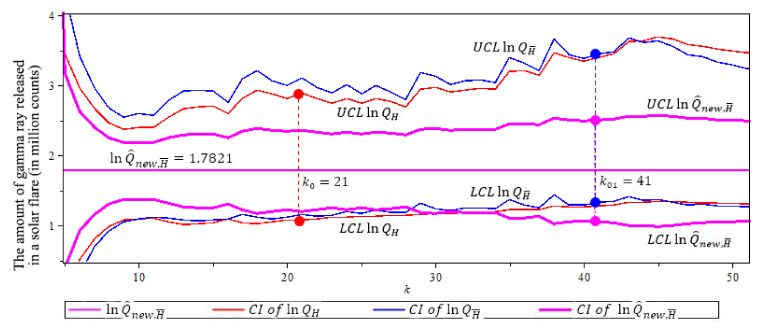
95% confidence interval of three ln-quantile estimators after threshold of 86 million counts for the gamma ray example. n=104, p=0.01. Note that $\ln{\hat{Q}}_{new,\bar{H}}$ (purple) has shortest CI with length 1.4451. (The solid circles “•” in the plot are the values of the quantile estimators at their optimal *k* level).

Figure 15 compares the confidence intervals of our ln-quantile estimators in (7), (12) and (19). This figure shows that the new quantile estimator $\ln{\hat{Q}}_{new,,\bar{H}}$ has the smallest confidence interval with length 1.4451, where we use $\hat{\alpha }=\hat{\rho }=-0.7269.$ The solid circles “•” in the plot are the values of the quantile estimators at their optimal *k* level.

In Table 12, we compare all four quantile estimators under $VaR_{0.05}$ and $VaR_{0.01}.$ Table 13 compares the size of confidence intervals of $\ln VaR_{0.01}$ and $VaR_{0.01}$ by three quantile estimators.

Table 13 shows that the new estimator has the shortest confidence interval, compared to ln$Q_{H}$, and ln$Q_{\bar{H}},$ with the highest efficiency of 1.6016.

#### 5.2.3. Summary

Based on Figure 15 and Table 13, we conclude that the new estimator $\ln{\hat{Q}}_{new,\bar{H}}$ in (19) is the best estimator for Gamma Ray example. We predict that $VaR_{0.01}$ is a gamma ray release of 1102.57 million counts, this is most likely an X rated solar flare. This is shown in Figure 13b.

## 6. Conclusions

Based on the studies in this paper, we conclude that:

1. High quantile and its CI estimation provides important information for risk management and for extreme event predictions.

2. Based on the theoretical and simulation results, the proposed new method for estimating confidence interval of high quantiles has advantages properties comparing with other existing methods. The estimation is consistent and stable with less error. The proposed method provides a useful computational algorithm to the readers.

3. The confidence interval of high quantile obtained by the new proposed method also has the highest efficiency compared to the existing methods, in terms of having the smallest size of confidence interval, and the highest probability coverage of the true quantile values in most cases.

4. Based on the analysis of the two real-world examples, flu in Canada and gamma ray from the solar flare, we can see that the new proposed method can be applied to many more fields, including other extreme events such as insurance claims, natural disasters, stock market predictions and pandemic disease monitoring.

## Figures and Tables

**Table 1:** The four ln-quantile estimators we use in simulations.

Quantile Estimators	Defined in	Tail Index Estimator
$\ln Q_{\hat{\gamma }=H}=\ln q_{H}$	(6)	*H* in (5)
$\ln Q_{H}$	(7)	*H* in (5)
$\ln{\tilde{Q}}_{\bar{H}}$	$\begin{array}{c} \ln Q_{\bar{H}}\;\mathrm{when}\;\rho \neq -1\;\mathrm{in}\;\left(12\right)\\ \ln{\bar{Q}}_{\bar{H}}\;\mathrm{when}\;\rho =-1\;\mathrm{in}\;\left(14\right) \end{array}$	$\bar{H}$ in (9)
$ln{\hat{Q}}_{new,\bar{H}}$	(19)	$\bar{H}$ in (9)

**Table 2:** Fréchet (0.25),N=500, β=0.5,ρ=−1. Mean, MSE, REFF of the lnVaR Estimators. The highest REFF values are in bold.

*n*		500	1000	2000	5000
$\ln{VaR}_{p},$ p=1/(2n)		1.7268	1.9002	2.0735	2.3026
$k_{0}$		126	200	318	585
$\hat{\alpha }\left(n\right)=LR$		1.1218	1.1400	0.4991	−0.0357
$\ln q_{H}$	Mean (MSE)	1.8526 (0.0429)	2.0038 (0.0300)	2.1657 (0.0228)	2.3755 (0.0147)
	REFF	1	1	1	1
$\ln Q_{H}$	Mean (MSE)	1.7906 (0.0219)	1.9540 (0.0154)	2.1239 (0.0115)	2.3431 (0.0074)
	REFF	1.4004	1.3933	1.4104	1.4125
$\ln{\bar{Q}}_{\bar{H}}$	Mean (MSE)	1.7092 (0.0206)	1.8849 (0.0141)	2.0764 (0.0111)	2.3073 (0.0072)
	REFF	1.4419	1.4576	1.4347	1.4257
$\ln{\hat{Q}}_{new,\bar{H}}$	Mean (MSE)	1.7185 (0.0095)	1.8791 (0.0065)	2.0716 (0.0051)	2.2798 (0.0044)
	REFF	**2.1252**	**2.1399**	**2.1139**	**1.8231**

**Table 3:** GPD (0.5), N=500, β=1,ρ=−0.5. Mean, MSE, REFF of the lnVaR estimators. The highest REFF values are in bold.

*n*		500	1000	2000	5000
$\ln{VaR}_{p},$ p=1/(2n)		4.1149	4.4710	4.8242	5.2883
$k_{0}$		34	48	68	107
$\hat{\alpha }\left(n\right)=\hat{\rho }$		−0.7512	−0.7482	−0.7427	−0.7244
$\ln q_{H}$	Mean (MSE)	4.7019 (0.6349)	4.9773 (0.4863)	5.3065 (0.4554)	5.7209 (0.3427)
	REFF	1	1	1	1
$\ln Q_{H}$	Mean (MSE)	4.2913 (0.2172)	4.6258 (0.1628)	4.9904 (0.1491)	5.4485 (0.1074)
	REFF	1.7159	1.7282	1.7478	1.7865
$\ln Q_{\bar{H}}$	Mean (MSE)	4.1140 (0.1654)	4.4801 (0.1267)	4.8656 (0.1166)	5.3434 (0.0825)
	REFF	1.9663	1.9591	1.9763	2.0379
$\ln{\hat{Q}}_{new,\bar{H}}$	Mean (MSE)	3.9076 (0.0779)	4.4239 (0.0233)	4.8674 (0.0241)	5.4359 (0.0382)
	REFF	**2.8657**	**4.5666**	**4.3428**	**2.9954**

**Table 4:** GPD(2), N=500, β=1,ρ=−2. Mean, MSE, REFF of the lnVaR estimators. The highest REFF values are in bold.

*n*		500	1000	2000	5000
$\ln{VaR}_{p},$ p=1/(2n)		13.1224	14.5087	15.8949	17.7275
$k_{0}$		170	269	515	1071
$\hat{\alpha }\left(n\right)=LR$		−2.7893	−2.8417	−2.8687	−3.1684
$\ln q_{H}$	Mean (MSE)	13.6276 (1.3232)	14.9415 (0.9745)	16.2733 (0.6548)	18.0099 (0.3833)
	REFF	1	1	1	1
$\ln Q_{H}$	Mean (MSE)	13.4502 (0.9965)	14.8004 (0.7283)	16.1618 (0.4779)	17.9283 (0.2804)
	REFF	1.1523	1.1567	1.2412	1.1693
$\ln Q_{\bar{H}}$	Mean (MSE)	13.1933 (0.8926)	14.5960 (0.6477)	15.9719 (0.4491)	17.7717 (0.2751)
	REFF	1.2175	1.2267	1.2075	1.1804
$\ln{\hat{Q}}_{new,\bar{H}}$	Mean (MSE)	13.0009 (0.6007)	14.4907 (0.3680)	15.8926 (0.3070)	17.6429 (0.2127)
	REFF	**1.4841**	**1.6274**	**1.4606**	**1.3426**

**Table 5:** N=500,n=1000, efficiencies of 95% CI for $\ln VaR_{0.01}$.

	CI of	at Optimal *k*	Length	${\mathrm{EFF}}_{\mathrm{length}}$	$\begin{array}{c} \mathrm{Probability}\\ \mathrm{Coverage} \end{array}$	${\mathrm{EFF}}_{P.C.}$
	$\ln Q_{H}$	$k_{0}^{Q_{H}}=$ 165	0.5142	1	94.2%	1
Fréchet (0.25)	$\ln{\bar{Q}}_{\bar{H}}$	$k_{01}=$ 395	0.3564	1.4517	96.7%	0.4706
	$\ln{\hat{Q}}_{new,\bar{H}}$	$k_{01}=$ 395	0.2668	1.9275	99.6%	0.1739
	$\ln Q_{H}$	$k_{0}^{Q_{H}}=$ 28	2.4922	1	47.4%	1
GPD(0.5)	$\ln Q_{\bar{H}}$	$k_{01}=$ 93	1.5204	1.6392	79.0%	2.9750
	$\ln{\hat{Q}}_{new,\bar{H}}$	$k_{01}=$ 93	0.7094	3.5130	99.6%	10.3478
	$\ln Q_{H}$	$k_{0}^{Q_{H}}=$ 270	3.4410	1	79.7%	1
GPD(2)	$\ln Q_{\bar{H}}$	$k_{01}=$ 511	2.7291	1.2609	83.2%	1.2966
	$\ln{\hat{Q}}_{new,\bar{H}}$	$k_{01}=$ 511	2.2511	1.5286	99.6%	3.3261

**Table 6:** The goodness-of-fit tests under the GPD model for the flu in Canada data.

	Goodness-of-Fit Tests					
	K-S Test		A-D Test		C-v-M Test	
	$\begin{array}{c} \mathrm{Test}\\ \mathrm{Statistics} \end{array}$	*p*-Value	$\begin{array}{c} \mathrm{Test}\\ \mathrm{Statistics} \end{array}$	p-Value	$\begin{array}{c} \mathrm{Test}\\ \mathrm{Statistics} \end{array}$	p-Value
${\hat{\gamma }}_{MLE}$	0.0628	0.6406	0.4475	0.8007	0.0621	0.8006

**Table 7:** AE and IE under the GPD model for the flu in Canada data by using ${\hat{\gamma }}_{MLE}$.

	Absolute Errors (AE)			Integrated Errors (IE)		
	$\begin{array}{c} r\mathrm{th}\;\mathrm{Highest}\;\mathrm{Amount}\\ \mathrm{of}\;\mathrm{Type}\;A\;\mathrm{Viruses} \end{array}$			$\begin{array}{c} r\mathrm{th}\;\mathrm{Highest}\;\mathrm{Amount}\\ \mathrm{of}\;\mathrm{Type}\;A\;\mathrm{Viruses} \end{array}$		
	r=12	r=56	r=111	r=12	r=56	r=111
${\hat{\gamma }}_{MLE}$	0.0450	0.0450	0.0628	0.0085	0.0071	0.0074

**Table 8:** Estimated $VaR_{0.05}$ and $VaR_{0.01}$ for the flu in Canada data. (Unit: Type A flu viruses).

Estimation	$\hat{\alpha }$	$\hat{\gamma }$	Mean	Median	${\mathrm{VaR}}_{0.05}$	${\mathrm{VaR}}_{0.01}$
$\ln Q_{H}$	N/A	H= 0.4370	3219.29	2257.03	4519.70	8159.10
$\ln Q_{\bar{H}}$	N/A	$\bar{H}\;=$ 0.3736	2989.93	2130.78	3736.80	6031.79
$\ln{\hat{Q}}_{new,\bar{H}}$	$\hat{\rho }\;=\;-0.7101$	$\bar{H}\;=$ 0.3736	2989.93	1690.07	2924.80	5499.85

**Table 9:** The 95% confidence interval for $\ln VaR_{0.01}$ and $VaR_{0.01}$.

$\begin{array}{c} \mathrm{Estimation}\\ \mathrm{Method} \end{array}$	*k*	LCL	$\ln{\mathrm{VaR}}_{0.01}$ (${\mathrm{VaR}}_{0.01})$	UCL	Length	EFF
$\ln Q_{H}$	${\hat{k}}_{0}=21$	0.6920	1.7312	2.1452	1.4531	1
($Q_{H})$		(3502.14)	(8159.10)	(11854.31)	(8352.17)	(1)
$\ln Q_{\bar{H}}$	${\hat{k}}_{01}=42$	0.7929	1.3814	1.9698	1.1770	1.2346
($Q_{\bar{H}})$		(3772.58)	(6031.78)	(10101.19)	(6328.21)	(1.3197)
$\ln Q_{new,\bar{H}}$	${\hat{k}}_{01}=42$	0.8724	1.2707	1.6690	0.7966	1.8242
($Q_{new,\bar{H}})$		(4006.07)	(5499.85)	(7724.49)	(3718.42)	(2.2462)

**Table 10:** Compare the goodness-of-fit tests under the GPD model for the gamma ray data.

	Goodness-of-Fit Tests					
	K-S Test		A-D Test		C-v-M Test	
	$\begin{array}{c} \mathrm{Test}\\ \mathrm{Statistics} \end{array}$	*p*-Value	$\begin{array}{c} \mathrm{Test}\\ \mathrm{Statistics} \end{array}$	p-Value	$\begin{array}{c} \mathrm{Test}\\ \mathrm{Statistics} \end{array}$	p-Value
${\hat{\gamma }}_{MLE}$	0.0697	0.5750	0.7276	0.5362	0.0991	0.5893

**Table 11:** AE and IE under the GPD model for the gamma ray data using ${\hat{\gamma }}_{MLE}$.

	Absolute Errors (AE)			Integrated Errors (IE)		
	*r*th Highest Gamma Ray Released			*r*th Highest Gamma Ray Released		
	r=11	r=53	r=104	r=11	r=53	r=104
${\hat{\gamma }}_{MLE}$	0.0359	0.0697	0.0697	0.0062	0.0092	0.0089

**Table 12:** Estimated $VaR_{0.05}$ and $VaR_{0.01}$ in the gamma ray example. (Unit: million counts).

$\begin{array}{c} \mathrm{Estimation}\\ \mathrm{Method} \end{array}$	$\hat{\alpha }$	$\hat{\gamma }$	Mean	Median	${\mathrm{VaR}}_{0.05}$	${\mathrm{VaR}}_{0.01}$
$\ln Q_{H}$	N/A	H=0.5324	451.82	315.27	867.12	1926.04
$\ln Q_{\bar{H}}$	N/A	$\bar{H}\;=\;$0.6517	577.22	232.28	742.01	1958.67
$\ln{\hat{Q}}_{new,\bar{H}}$	$\hat{\rho }\;=\;-$0.7269	$\bar{H}\;=\;$0.6517	577.22	189.35	441.60	1102.57

**Table 13:** The 95% confidence interval of $\ln VaR_{0.01}t.$ and $VaR_{0.01}.$

$\begin{array}{c} \mathrm{Estimation}\\ \mathrm{Method} \end{array}$	*k*	LCL	$\ln{\mathrm{VaR}}_{0.01}$ (${\mathrm{VaR}}_{0.01})$	UCL	Length	EFF
$\ln Q_{H}$	${\hat{k}}_{0}\;=\;21$	1.0807	2.3755	2.8864	1.8057	1
($Q_{H})$		(590.12)	(1926.04)	(3153.18)	(2563.06)	(1)
$\ln Q_{\bar{H}}$	${\hat{k}}_{01}\;=\;41$	1.3367	2.3930	3.4494	2.1128	0.8547
($Q_{\bar{H}})$		(737.14)	(1958.67)	(5471.71)	(4734.56)	(0.5414)
$\ln Q_{new,\bar{H}}$	${\hat{k}}_{01}\;=\;41$	1.0595	1.7821	2.5047	1.4451	1.2495
($Q_{new,\bar{H}})$		(579.55)	(1102.57)	(2179.85)	(1600.30)	(1.6016)

## Data Availability

Publicly available datasets were analyzed in this study. The datasets can be found here: http://hesperia.gsfc.nasa.gov/fermi/gbm/qlook/fermi_gbm_flare_list.txt [31] and https://www.who.int/influenza/gisrs_laboratory/flunet/en [28].

## Appendix A. Proofs of Theorems 1 and 2

**Lemma** **1.**
*The sum of* $Cov\left[\ln{\bar{Q}}_{\bar{H}}^{\left(p\right)}\left(i\right),\ln{\bar{Q}}_{\bar{H}}^{\left(p\right)}\left(j\right)\right],$ i≠j,i,j=1,...,n−1, *satisfy the inequality*

(A1) $$\underset{i\neq j}{\sum^{n-1}\sum^{n-1}}Cov\left[\ln{\bar{Q}}_{\bar{H}}^{\left(p\right)}\left(i\right),\ln{\bar{Q}}_{\bar{H}}^{\left(p\right)}\left(j\right)\right]\leq \;w\;\underset{i\neq j}{\sum^{n-1}\sum^{n-1}}\sqrt{\left(Var\left(\ln{\bar{Q}}_{\bar{H}}^{\left(p\right)}\left(i\right)\right)Var\left(\ln{\bar{Q}}_{\bar{H}}^{\left(p\right)}\left(j\right)\right)\right)},$$

*where w is a weight*

$$w=\max_{i\neq j}\rho_{ij}^{+},\;0\leq w\leq 1,\;\mathrm{where}\;\rho_{ij}^{+}=\left|\rho_{ij}^{}\right|,\;0\leq \rho_{ij}^{+}\leq 1;$$

$\rho_{ij}^{}$ *is correlation coefficient of* $\ln{\bar{Q}}_{\bar{H}}^{\left(p\right)}\left(i\right)$ *and* $\ln{\bar{Q}}_{\bar{H}}^{\left(p\right)}\left(j\right)$

$$\rho_{ij}^{}=\frac{Cov\left[\ln{\bar{Q}}_{\bar{H}}^{\left(p\right)}\left(i\right),\ln{\bar{Q}}_{\bar{H}}^{\left(p\right)}\left(j\right)\right]}{\sqrt{\left(Var\left(\ln{\bar{Q}}_{\bar{H}}^{\left(p\right)}\left(i\right)\right)Var\left(\ln{\bar{Q}}_{\bar{H}}^{\left(p\right)}\left(j\right)\right)\right)}},\;i\neq j,\;\;i,\;j=1,...,n-1,\;\;-1\leq \rho_{ij}^{}\leq 1.$$

**Proof** **of** **Lemma 1.** For each pair (i,j), i≠j,i,j=1,...,n−1

$$Cov\left[\ln{\bar{Q}}_{\bar{H}}^{\left(p\right)}\left(i\right),\ln{\bar{Q}}_{\bar{H}}^{\left(p\right)}\left(j\right)\right]=\rho_{ij}^{}\sqrt{\left(Var\left(\ln{\bar{Q}}_{\bar{H}}^{\left(p\right)}\left(i\right)\right)Var\left(\ln{\bar{Q}}_{\bar{H}}^{\left(p\right)}\left(j\right)\right)\right)}$$

then we have a boundary if we only add the positive $\rho_{ij}^{+}$ terms in the following summation

$$\begin{array}{ccc} \underset{i\neq j}{\sum^{n-1}\sum^{n-1}}Cov\left[\ln{\bar{Q}}_{\bar{H}}^{\left(p\right)}\left(i\right),\ln{\bar{Q}}_{\bar{H}}^{\left(p\right)}\left(j\right)\right] & = & \underset{i\neq j}{\sum^{n-1}\sum^{n-1}}\rho_{ij}\sqrt{\left(Var\left(\ln{\bar{Q}}_{\bar{H}}^{\left(p\right)}\left(i\right)\right)Var\left(\ln{\bar{Q}}_{\bar{H}}^{\left(p\right)}\left(j\right)\right)\right)}\\ & \leq & \underset{i\neq j}{\sum^{n-1}\sum^{n-1}}\rho_{ij}^{+}\sqrt{\left(Var\left(\ln{\bar{Q}}_{\bar{H}}^{\left(p\right)}\left(i\right)\right)Var\left(\ln{\bar{Q}}_{\bar{H}}^{\left(p\right)}\left(j\right)\right)\right)}\\ & \leq & w\underset{i\neq j}{\sum^{n-1}\sum^{n-1}}\sqrt{\left(Var\left(\ln{\bar{Q}}_{\bar{H}}^{\left(p\right)}\left(i\right)\right)Var\left(\ln{\bar{Q}}_{\bar{H}}^{\left(p\right)}\left(j\right)\right)\right)}.\;□ \end{array}$$

**Lemma** **2.**
*Under conditions (C1) and (C2), for* $\ln{\bar{Q}}_{\bar{H}}^{\left(p\right)}\left(k\right)$ *in (14) by use Theorem 5.1, formula (5.2) in Gomes and Pestana (2007, p.285 [17]), as* n→∞,

$$\frac{\sqrt{k}}{\ln\left(\frac{k}{np}\right)}\left(\ln{\bar{Q}}_{\bar{H}}^{\left(p\right)}\left(k\right)-\ln VaR_{p}\right)\overset{d}{\;\vec{n\to \infty }\;}Normal\left(0,\;\gamma^{2}\right),$$

*then the asymptotic expected value and variance are*

$$E\left(\ln{\bar{Q}}_{\bar{H}}^{\left(p\right)}\left(k\right)-\ln VaR_{p}\right)\approx 0,\;as\;n\to \infty ;$$

(A2) $$Var\left(\ln{\bar{Q}}_{\bar{H}}^{\left(p\right)}\left(k\right)\right)\approx \gamma^{2}\frac{{\left(\ln\left(\frac{k}{np}\right)\right)}^{2}}{k},\;as\;n\to \infty .$$

**Proof** **of** **Theorem 1.** Under conditions (C1) and (C2), in the Hall-Welsh class of models in (6), where $\bar{H}$ is in (8) with conditions

$$\sqrt{k}\left({\bar{H}}_{\beta ,\rho }\left(k\right)-\gamma \right)\overset{d}{\underset{n\to \infty }{⟶}}Normal\left(0,\gamma \right),$$

and

$${\bar{H}}_{\hat{\beta },\hat{\rho }}\left(k\right)\overset{d}{\underset{n\to \infty }{⟶}}=\gamma +\frac{\gamma }{\sqrt{k}}V_{k}+o_{p}\left(A\left(\frac{n}{k}\right)\right),$$

where $V_{k}$ is an asymptotic standard normal random variable. By Schwartz inequality and Lemma 1 formula (A1), sinece α is a contant in (19), based on asympototic properties of $C_{p}\left(k;\hat{\beta },\hat{\rho }\right)$ in (13) (Gomes and Pestana, 2007, p.286 [17]), we have that

$$\begin{array}{ll} & Var\left(\ln{\hat{Q}}_{New,\bar{H}}^{\left(p\right)}\right)\\ = & Var\left[\left(\frac{1}{n-1}\sum_{k=1}^{n-1}\ln{\bar{Q}}_{\bar{H}}^{\left(p\right)}\left(k\right)\right)\right]\\ = & \frac{1}{{\left(n-1\right)}^{2}}\left\{\sum_{k=1}^{n-1}Var\left[\left(\ln{\bar{Q}}_{\bar{H}}^{\left(p\right)}\left(k\right)\right)\right]+\underset{i\neq j}{\sum^{n-1}\sum^{n-1}}Cov\left[\ln{\bar{Q}}_{\bar{H}}^{\left(p\right)}\left(i\right),\ln{\bar{Q}}_{\bar{H}}^{\left(p\right)}\left(j\right)\right]\right\}\\ = & \frac{1}{{\left(n-1\right)}^{2}}\left\{\sum_{k=1}^{n-1}Var\left[\left(\ln{\bar{Q}}_{\bar{H}}^{\left(p\right)}\left(k\right)\right)\right]+\underset{i\neq j}{\sum^{n-1}\sum^{n-1}}\rho_{ij}\sqrt{\left(Var\left(\ln{\bar{Q}}_{\bar{H}}^{\left(p\right)}\left(i\right)\right)Var\left(\ln{\bar{Q}}_{\bar{H}}^{\left(p\right)}\left(j\right)\right)\right)}\right\}\\ \leq & \frac{1}{{\left(n-1\right)}^{2}}\left\{\sum_{k=1}^{n-1}Var\left[\left(\ln{\bar{Q}}_{\bar{H}}^{\left(p\right)}\left(k\right)\right)\right]+w\;\sum_{i\neq j}^{n-1}\sum^{n-1}\sqrt{\left(Var\left(\ln{\bar{Q}}_{\bar{H}}^{\left(p\right)}\left(i\right)\right)Var\left(\ln{\bar{Q}}_{\bar{H}}^{\left(p\right)}\left(j\right)\right)\right)}\right\}. \end{array}$$

Therefore when *n* is large enough, use Lemma 2, formula (A2),

$$Var\left(\ln{\bar{Q}}_{\bar{H}}^{\left(p\right)}\left(k\right)\right)\approx \gamma^{2}\frac{{\left(\ln\left(\frac{k}{np}\right)\right)}^{2}}{k},\;as\;n\to \infty ;$$

and

$$E\left(\ln{\hat{Q}}_{New,\bar{H}}^{\left(p\right)}-\ln VaR_{p}\right)\approx 0,\;as\;n\to \infty .$$

we can have the following approximate relation

$$\begin{array}{ccc} Var\left(\ln{\hat{Q}}_{New,\bar{H}}^{\left(p\right)}\right) & \leq & \frac{1}{{\left(n-1\right)}^{2}}\left[\sum_{k=1}^{n-1}\gamma^{2}\frac{{\left[\ln\left(\frac{k}{np}\right)\right]}^{2}}{k}+w\sum_{i\neq j}^{n-1}\sum^{n-1}\gamma^{2}\sqrt{\frac{{\left[\ln\left(\frac{i}{np}\right)\right]}^{2}}{i}}\sqrt{\frac{{\left(\ln\left(\frac{j}{np}\right)\right)}^{2}}{j}}\right]\\ & = & \frac{\gamma^{2}}{{\left(n-1\right)}^{2}}\left[\sum_{k=1}^{n-1}\frac{{\left[\ln\left(\frac{k}{np}\right)\right]}^{2}}{k}+w\;\sum_{i\neq j}^{n-1}\sum^{n-1}\left[\frac{\ln\left(\frac{i}{np}\right)\ln\left(\frac{j}{np}\right)}{\sqrt{i\cdot j}}\right]\right],\;n\to \infty . \end{array}$$

this proved (21). Therefore, we have the asymptotic normal distribution in (20)

(A3) $$\left(\ln{\hat{Q}}_{New,\bar{H}}^{\left(p\right)}-\ln VaR_{p}\right)\overset{d}{\underset{n\to \infty }{⟶}}Normal\left(0,\frac{\gamma^{2}}{{\left(n-1\right)}^{2}}\left[\sum_{k=1}^{n-1}\frac{{\left[\ln\frac{k}{np}\right]}^{2}}{k}+w\sum_{i<j}^{n-1}\sum^{n-1}\left[\frac{\ln\left(\frac{i}{np}\right)\ln\left(\frac{j}{np}\right)}{\sqrt{i\cdot j}}\right]\right]\right).$$

Furthermore, we use (21) and (A2), we obtain (22) as

$$\begin{array}{ccc} EFF{\left(\ln{\hat{Q}}_{New,\bar{H}}^{\left(p\right)}\right)}_{\ln{\hat{Q}}_{\bar{H}}^{\left(p\right)}\left(k\right)} & = & \frac{Var\left(\ln{\bar{Q}}_{\bar{H}}^{\left(p\right)}\left(k\right)\right)}{Var\left(\ln{\hat{Q}}_{New,\bar{H}}^{\left(p\right)}\right)}\\ & \geq & \frac{{\left(n-1\right)}^{2}\frac{{\left(\ln\left(\frac{k}{np}\right)\right)}^{2}}{k}}{\sum_{k=1}^{n-1}\frac{{\left[\ln\left(\frac{k}{np}\right)\right]}^{2}}{k}+w\sum^{n-1}\sum^{n-1}\left[\frac{\ln\left(\frac{i}{np}\right)\ln\left(\frac{j}{np}\right)}{\sqrt{i•j}}\right]},\;n\to \infty .\;□ \end{array}$$

**Proof** **of** **Theorem 2.** Under conditions (C1) and (C2), Use $Var\left(\ln{\hat{Q}}_{New,\bar{H}}^{\left(p\right)}\right)$ in Theorem 1, Formula (22), and $z_{\alpha /2}=-z_{1-\alpha /2},$ with

$$b3=\frac{z_{1-\alpha /2}}{n-1}\sqrt{\sum_{k=1}^{n-1}\frac{{\left[\ln\left(\frac{k}{np}\right)\right]}^{2}}{k}+w\sum_{i\neq j}^{n-1}\sum^{n-1}\left[\frac{\ln\left(\frac{i}{np}\right)\ln\left(\frac{j}{np}\right)}{\sqrt{i\cdot j}}\right]},\;UCL_{\bar{H}}\left(k\right)=\frac{\bar{H}}{1-\;\frac{z_{1-\alpha /2}}{\sqrt{k}}}.$$

we have

$$Z=\frac{\sqrt{n-1}}{\gamma \sqrt{\sum_{k=1}^{n-1}\frac{{\left[\ln\left(\frac{k}{np}\right)\right]}^{2}}{k}+w\underset{i\neq j}{\sum^{n-1}\sum^{n-1}}\left[\frac{\ln\left(\frac{i}{np}\right)\ln\left(\frac{j}{np}\right)}{\sqrt{i•j}}\right]}}\left(\ln{\hat{Q}}_{New,\bar{H}}^{\left(p\right)}-\ln VaR_{p}\right)\;\overset{d}{\vec{n\to \infty }}\;Normal\left(0,1\right),$$

therefore approximately

$$P\left\{-z_{1-\alpha /2}\leq \frac{n-1}{\gamma \sqrt{\sum_{k=1}^{n-1}\frac{{\left[\ln\left(\frac{k}{np}\right)\right]}^{2}}{k}+\underset{i\neq j}{w\sum^{n-1}\sum^{n-1}}\left[\frac{\ln\left(\frac{i}{np}\right)\ln\left(\frac{j}{np}\right)}{\sqrt{i•j}}\right]}}\left(\ln{\hat{Q}}_{New,\bar{H}}^{\left(p\right)}-\ln VaR_{p}\right)\leq z_{1-\alpha /2}\right\}$$

=1−α, then

$$P\left\{-\gamma b3\leq \left(\ln{\hat{Q}}_{New,\bar{H}}^{\left(p\right)}-\ln VaR_{p}\right)\leq \gamma b3\right\}=1-\alpha$$

$$P\left\{-\ln{\hat{Q}}_{New,\bar{H}}^{\left(p\right)}-\gamma b3\leq -\ln VaR_{p}\leq -\ln{\hat{Q}}_{New,\bar{H}}^{\left(p\right)}+\gamma b3\right\}=1-\alpha ,$$

and using $\hat{\gamma }=\bar{H}\left(k\right)\leq UCL_{\bar{H}}\left(k\right),$ to guarantee a coverage probability of at least (1−α)100%, we have

$$P\left\{\ln{\hat{Q}}_{New,\bar{H}}^{\left(p\right)}-UCL_{\bar{H}}\left(k\right)b3\leq \ln VaR_{p}\leq \ln{\hat{Q}}_{New,\bar{H}}^{\left(p\right)}+UCL_{\bar{H}}\left(k\right)b3\right\}\geq 1-\alpha .\;□$$
